# Supplementary material for: Osteology of an exceptionally well-preserved tapejarid skeleton from Brazil: Revealing the anatomy of a curious pterodactyloid clade
Source: PLoS One. 2021 Aug 25;16(8):e0254789. doi: 10.1371/journal.pone.0254789 (PMC8386889; doi:10.1371/journal.pone.0254789)
Supplement: Attachment — Submitted filename: PONE-D-20-38270_AA.pdf [file pone.0254789.s003.pdf]

PLOS ONE

Osteology of an exceptionally well-preserved tapejarid skeleton from Brazil: revealing the anatomy of a curious pterodactyloid clade

--Manuscript Draft--

Manuscript Number:	PONE-D-20-38270
Article Type:	Research Article
Full Title:	Osteology of an exceptionally well-preserved tapejarid skeleton from Brazil: revealing the anatomy of a curious pterodactyloid clade
Short Title:	Description of a nearly complete Tupandactylus navigans
Corresponding Author:	Victor Beccari, Bachelor of Biological Sciences<br>University of Sao Paulo: Universidade de Sao Paulo<br>São Paulo, São Paulo BRAZIL
Keywords:	Pterosauria, Pterodactyloidea, Tapejaridae, Tupandactylus navigans, Lower Cretaceous, Santana Group
Abstract:	A remarkably well-preserved, almost completely articulated new specimen (GP/2E 9266) of Tupandactylus navigans is here described for the Lower Cretaceous Crato Formation of Brazil. The new specimen comprises an almost complete skeleton with remarkable preservation of soft tissues associated with both the skull and post-cranium, which makes it the most complete Brazilian tapejarid known thus far. CT-Scanning was performed to allow the assessment of bones still covered by sediment. The specimen can be assigned to T. navigans due to its vertical supra premaxillary bony process and short and rounded parietal crest. It also bears the largest dentary crest among Tapejarine pterosaurs and a notarium, which is absent in other members of the clade. The new specimen is a subadult individual with overall similar cranial proportions as Tupandactylus imperator. This is the first time the postcranial remains of T. navigans are described, being also an unprecedented record of an articulated tapejarid skeleton from the Araripe Basin.
Order of Authors:	Victor Beccari, Bachelor of Biological Sciences
	Felipe Lima Pinheiro
	Ivan Nunes
	Luiz Eduardo Anelli
	Octávio Mateus
	Fabiana Rodrigues Costa
Additional Information:	
**Question**	**Response**
**Financial Disclosure**<br><br>Enter a financial disclosure statement that describes the sources of funding for the work included in this submission. Review the submission guidelines for detailed requirements. View published research articles from *PLOS ONE* for specific examples.<br><br>This statement is required for submission and **will appear in the published article** if the submission is accepted. Please make sure it is accurate.	FLP is supported by grants from Conselho Nacional de Desenvolvimento Científico e Tecnológico (CNPq process numbers 407969/2016-0, 305758/2017-9) and Fundacão de Amparo à Pesquisa do Estado do Rio Grande do Sul (FAPERGS process number 16/2551-0000271-1). OM is supported by grants from GeoBioTec-GeoBioSciences, GeoTechnologies and GeoEngineering NOVA [GeoBioCiências, GeoTecnologias e GeoEngenharias], grant UIDB/04035/2020 by the Fundação para a Ciência e Tecnologia. FRC is supported by Conselho Nacional de Desenvolvimento Científico e Tecnológico (CNPq) for support (grant No. 421772/2018-2).

Powered by Editorial Manager® and ProduXion Manager® from Aries Systems Corporation

**Unfunded studies**<br>Enter: *The author(s) received no specific funding for this work.*<br><br>**Funded studies**<br>Enter a statement with the following details:<br>• Initials of the authors who received each award<br>• Grant numbers awarded to each author<br>• The full name of each funder<br>• URL of each funder website<br>• Did the sponsors or funders play any role in the study design, data collection and analysis, decision to publish, or preparation of the manuscript?<br>• **NO** - Include this sentence at the end of your statement: *The funders had no role in study design, data collection and analysis, decision to publish, or preparation of the manuscript.*<br>• **YES** - Specify the role(s) played.<br><br>* typeset	
**Competing Interests**<br><br>Use the instructions below to enter a competing interest statement for this submission. On behalf of all authors, disclose any competing interests that could be perceived to bias this work—acknowledging all financial support and any other relevant financial or non-financial competing interests.<br><br>This statement **will appear in the published article** if the submission is accepted. Please make sure it is accurate. View published research articles from *PLOS ONE* for specific examples.	NO authors have competing interests

**NO authors have competing interests**<br><br>Enter: *The authors have declared that no competing interests exist.*<br><br>**Authors with competing interests**<br><br>Enter competing interest details beginning with this statement:<br><br>*I have read the journal's policy and the authors of this manuscript have the following competing interests: [insert competing interests here]*<br><br>* typeset	
**Ethics Statement**<br><br>Enter an ethics statement for this submission. This statement is required if the study involved:<br><br>• Human participants<br>• Human specimens or tissue<br>• Vertebrate animals or cephalopods<br>• Vertebrate embryos or tissues<br>• Field research<br><br>Write "N/A" if the submission does not require an ethics statement.<br><br>General guidance is provided below. Consult the submission guidelines for detailed instructions. **Make sure that all information entered here is included in the Methods section of the manuscript.**	N/A

Powered by Editorial Manager® and ProduXion Manager® from Aries Systems Corporation

**Format for specific study types**<br><br>**Human Subject Research (involving human participants and/or tissue)**<br>• Give the name of the institutional review board or ethics committee that approved the study<br>• Include the approval number and/or a statement indicating approval of this research<br>• Indicate the form of consent obtained (written/oral) or the reason that consent was not obtained (e.g. the data were analyzed anonymously)<br><br>**Animal Research (involving vertebrate animals, embryos or tissues)**<br>• Provide the name of the Institutional Animal Care and Use Committee (IACUC) or other relevant ethics board that reviewed the study protocol, and indicate whether they approved this research or granted a formal waiver of ethical approval<br>• Include an approval number if one was obtained<br>• If the study involved *non-human primates*, add *additional details* about animal welfare and steps taken to ameliorate suffering<br>• If anesthesia, euthanasia, or any kind of animal sacrifice is part of the study, include briefly which substances and/or methods were applied<br><br>**Field Research**<br><br>Include the following details if this study involves the collection of plant, animal, or other materials from a natural setting:<br>• Field permit number<br>• Name of the institution or relevant body that granted permission	
**Data Availability**<br><br>Authors are required to make all data underlying the findings described fully available, without restriction, and from the time of publication. PLOS allows rare exceptions to address legal and ethical concerns. See the PLOS Data Policy and FAQ for detailed information.	Yes - all data are fully available without restriction

Powered by Editorial Manager® and ProduXion Manager® from Aries Systems Corporation

A Data Availability Statement describing where the data can be found is required at submission. Your answers to this question constitute the Data Availability Statement and **will be published in the article**, if accepted.<br><br>**Important:** Stating 'data available on request from the author' is not sufficient. If your data are only available upon request, select 'No' for the first question and explain your exceptional situation in the text box.<br><br>Do the authors confirm that all data underlying the findings described in their manuscript are fully available without restriction?	
**Describe where the data may be found in full sentences. If you are copying our sample text, replace any instances of XXX with the appropriate details.**<br><br>• If the data are **held or will be held in a public repository**, include URLs, accession numbers or DOIs. If this information will only be available after acceptance, indicate this by ticking the box below. For example: *All XXX files are available from the XXX database (accession number(s) XXX, XXX.*).<br>• If the data are all contained **within the manuscript and/or Supporting Information files**, enter the following: *All relevant data are within the manuscript and its Supporting Information files.*<br>• If neither of these applies but you are able to provide **details of access elsewhere**, with or without limitations, please do so. For example:<br><br>*Data cannot be shared publicly because of [XXX]. Data are available from the XXX Institutional Data Access / Ethics Committee (contact via XXX) for researchers who meet the criteria for access to confidential data.*<br><br>*The data underlying the results presented in the study are available from (include the name of the third party*	The Supplemental Files include the dataset adopted for the phylogenetic analysis The specimen described in this study is stored in the collection of the Coleção de Paleontologia Sistemática of the Geosciences Institute of Universidade de São Paulo under reference number: GP/2E 9266.<br><br>The 3D models will be available at MorphoMuseuM

and contact information or URL).<br>• This text is appropriate if the data are owned by a third party and authors do not have permission to share the data.<br><br>* typeset	
Additional data availability information:	Tick here if the URLs/accession numbers/DOIs will be available only after acceptance of the manuscript for publication so that we can ensure their inclusion before publication.

 Click here to access/download;Manuscript;Manuscript.docx

Osteology of an exceptionally well-preserved tapejarid skeleton from Brazil: revealing the anatomy of a curious pterodactyloid clade

Victor Beccari[1,2,3], Felipe L. Pinheiro[4], Ivan Nunes[5], Luiz Eduardo Anelli[6], Octávio Mateus[2,3], Fabiana R. Costa[7]

[1] Instituto de Biociências, Universidade de São Paulo, Campus São Paulo, Rua do Matão, Tv. 14 - Butantã, 05508-090, São Paulo, SP, Brazil

[2] GeoBioTec, Department of Earth Sciences, Faculdade de Ciências e Tecnologia, FCT, Universidade Nova de Lisboa, 2829-516 Caparica, Portugal

[3] Museu da Lourinhã, Lourinhã, Portugal

[4] Laboratório de Paleobiologia, Universidade Federal do Pampa, Campus São Gabriel, Av. Antônio Trilha, 1847, Centro, 97300-162, São Gabriel, RS, Brazil

[5] Instituto de Biociências, Laboratório de Herpetologia (LHERP), Universidade Estadual Paulista, Campus do Litoral Paulista, Praça Infante Don Henrique S/N,11.330-900, São Vicente, SP, Brazil

[6] Instituto de Geociências, Universidade de São Paulo, Campus São Paulo, Rua do Lago, 562, Cidade Universitária, 05508-080, São Paulo, SP, Brazil

[7] Centro de Ciências Naturais e Humanas, Laboratório de Paleontologia de Vertebrados e Comportamento Animal (LAPC), Universidade Federal do ABC, Campus São Bernardo do Campo, Rua São Paulo S/N, Jardim Antares, São Bernardo do Campo, SP, Brazil

Abstract

A remarkably well-preserved, almost completely articulated new specimen (GP/2E 9266) of *Tupandactylus navigans* is here described for the Lower Cretaceous Crato Formation of Brazil. The new specimen comprises an almost complete skeleton with remarkable preservation of soft tissues associated with both the skull and post-cranium, which makes it the most complete Brazilian tapejarid known thus far. CT-Scanning was performed to allow the assessment of bones still covered by sediment. The specimen can be assigned to *T. navigans* due to its vertical supra premaxillary bony process and short and rounded parietal crest. It also bears the largest dentary crest among Tapejarine pterosaurs and a notarium, which is absent in other members of the clade. The new specimen is a subadult individual with overall similar cranial proportions as *Tupandactylus imperator*. This is the first time the postcranial remains of *T. navigans* are described, being also an unprecedented record of an articulated tapejarid skeleton from the Araripe Basin.

Key words: Pterosauria, Pterodactyloidea, Tapejaridae, *Tupandactylus navigans*, Lower Cretaceous, Santana Group

Introduction

The pterosaur clade Tapejaridae was a major component of Lower Cretaceous continental faunas, achieving a widespread distribution in Gondwana and Eurasia (e.g. [1-3]). Tapejarids are characteristic for their edentulous jaws, often huge cranial crests, and for sometimes being associated with herbivorous feeding habits [1-2]. In Brazil, tapejarids are among the most abundant and diverse pterosaur taxa, being recovered from the Crato and Romualdo *Lagerstätten* (Araripe Basin, Northeastern part of the country) and from the desertic environments of the Goiô-Erê Formation (Paraná Basin, Southern Brazil) [4].

Most Brazilian tapejarids are known from isolate skulls or partial skeletons, with the exceptions of *Caiuajara dobruskii* and *Tapejara wellnhoferi*, from which several disarticulated specimens were recovered [4-5]. Up to now, the most complete tapejarid specimens were found in the Lower Cretaceous of China [6], but their anatomy has not yet been described in detail.

The genus *Tupandactylus*, perhaps the most impressive tapejarid known thus far due to its large soft-tissue sagittal crest, is comparatively abundant in Crato Formation limestones, with several specimens deposited in public and private collections (e.g. [7-9]). However, both *Tupandactylus* species — *T. imperator* [7] and *T. navigans* [8] — are known solely from isolated skulls [9].

Because the typical Crato Formation preservation hinders a complete preparation and isolation of bones, most pterosaur specimens from this unit were described using solely their superficially exposed features. Here we describe a nearly complete, almost fully articulated *T. navigans* skeleton (GP/2E 9266) with aid of CT-Scanning. The specimen was intercepted during a police raid at Santos Harbour, São Paulo State, and confiscated together with several other exceptionally well-preserved fossils now housed at Universidade de São Paulo (USP). Apart from presenting the first postcranial material unambiguously assigned to *Tupandactylus*, the new specimen is indeed the best-preserved tapejarid skeleton known thus far, shedding new light on the anatomic information for this pterodactyloid clade.

Geological Setting

Specimen GP/2E 9266 is preserved in six perfectly complementary yellowish limestone slabs, which fit together by rectilinear cuts. The cutting pattern reflects a typical procedure of quarryman to extract paving stones from Crato Formation outcrops. Albeit the exact

locality and horizon from where GP/2E 9266 was recovered are unknown, we secure its provenance from Crato Formation for two main reasons: first of all, the lithology of the embedding matrix perfectly fits the biomicritic laminated limestone beds of Crato Formation, being commercial exploitation a common practice in those deposits; secondly, all *Tupandactylus navigans* specimens previously reported come from Crato Fm. outcrops [8]. Among the sedimentary fill of the Araripe Basin (Northeastern Brazil), the Crato Formation rivals the younger Romualdo Formation in abundance and exceptional preservation of their fossil yielding. Regarded as Aptian in age, the Crato Fm. crops out following a mainly N-SE belt in the northern scarps of the Araripe *plateau* [10]. The genesis of Crato Formation laminated limestones is presumably related to authigenic carbonate precipitation and deposition following seasonal phytoplankton blooms or seasonal salinity fluctuations caused by evaporation [11-12]. Crato Formation carbonates were deposited in a quiet and protected environment, presenting evidence of strong chemocline, especially concerning salinity and oxygen concentration. The abundance of freshwater parautochthonous fauna (as Ephemeroptera larvae and anurans) in association to halite pseudomorphs indicates fresh shallow waters above an at least episodic hypersaline bottom. Similarly, the absence of benthic fauna and bioturbated sediments indicate that deep waters were anoxic [11-13].

Materials and Methods

Material

The specimen is housed at Laboratório de Paleontologia Sistemática of the Instituto de Geociências at Universidade de São Paulo (São Paulo, Brazil) under the collection number GP/2E 9266. It is preserved in six limestone slabs, four large square-cut plates comprising most the skeletal elements and soft-tissue crest (slabs 1 to 4, from upper left to bottom right), and two smaller rectangular-cut ones (slabs 5 and 6, bottom left to right).

When joint together these slabs perfectly tie all parts and the bones that had their pieces separated by these cuts. The specimen presents exquisite preservation of soft-tissue elements, and most of the preparation was done before its acquisition. Both left square-cut slabs (slabs 1 and 3) have been broken and were then rejoined with thin metal bars before the acquisition of the material. The preserved skeletal elements show different degrees taphonomic distortion (Table 1).

Table 1 – *Tupandactylus navigans* GP/2E 9266 preserved elements with comments on the preservation.

Bone(s)	State of preservation	Comments
Cranial bones	Varies, mainly laterally compressed	Most bones preserved; premaxillomaxilla divided in slabs 1 and 3; parietal crest fragmented
Mandible	Laterally compressed	Right mandibular ramus ventrally deflected
Hyoids	Preserved with minor distortion	Three ceratobranchials
Non-ossified tissue	Impression	Rhamphotheca present; sagittal crest divided into slabs 1, 2 and 3; dorsalmost margin of sagittal crest missing
Cervical vertebrae	Preserved with minor distortion	Nine cervical vertebrae preserved; atlantoaxis fused; cervical vertebrae 8 and 9 divided into slabs 3 and 4
Notarium	Laterally compressed	Composed by five dorsal vertebrae; left surface weathered

Dorsal vertebrae	Laterally compressed	Five free dorsal vertebrae; left surface weathered
Sacral vertebrae	Laterally compressed	Five fused sacral vertebrae; greatly weathered
Caudal vertebrae	Preserved with minor distortion	Five caudal vertebrae preserved
Dorsal ribs	Preserved with minor distortion	Nine dorsal ribs preserved; mainly fragmented
Scapulocoracoid (l)	Preserved with minor distortion	-
Scapulocoracoid (r)	Preserved with minor distortion	Divided into slabs 3 and 4
Sternum	Dorsoventrally compressed	Divided into slabs 3 and 4
Sacrum (l)	Preserved with minor distortion	Missing prepubis
Humerus (l)	Dorsoventrally compressed	Divided into slabs 4 and 6; highly flattened
Humerus (r)	Preserved with minor distortion	-
Ulna (l)	Dorsoventrally compressed	Divided into slabs 4 and 6; highly flattened
Ulna (r)	Dorsoventrally compressed	Proximally retains its form

Radius (l)	Dorsoventrally compressed	Divided into slabs 4 and 6; highly flattened
Radius (r)	Dorsoventrally compressed	Flattened
Carpals (l)	Preserved with minor distortion	3 carpal elements preserved
Carpals (r)	Laterally compressed	3 carpal elements and pteroid preserved
Metacarpals I-III (l)	Preserved with minor distortion	Distally articulated
Metacarpals I-III (r)	Preserved with minor distortion	Proximally articulated
Metacarpal IV (l)	Dorsoventrally compressed	Missing distal articulation surface
Metacarpal IV (r)	Dorsoventrally compressed	Divided into slabs 3 and 4
Manus (l)	Preserved with minor distortion	Articulated elements; complete
Manus (r)	Preserved with minor distortion	Disarticulated elements, divided into slabs 3 and 4
Wing phalanxes (l)	Preserved with minor distortion	Articulated elements; divided into slabs 3, 4 and 5; missing fourth wing phalanx
Wing phalanxes (r)	Preserved with minor distortion	Articulated elements; minor fractures
Femur (l)	Laterally compressed	Missing femoral head; distally retains its form

Femur (r)	Preserved with minor distortion	-
Tibia and fibula (l)	Laterally compressed	-
Tibia and fibula (r)	Laterally compressed	Distally retains its form
Tarsals (l)	Preserved with minor distortion	Three elements preserved
Tarsals (r)	Preserved with minor distortion	Three elements preserved
Metatarsals (l)	Preserved with minor distortion	Four elements preserved; fifth metatarsal absent, divided into slabs 4 and 6
Metatarsals (r)	Preserved with minor distortion	Four elements preserved; fifth metatarsal absent
Pes (l)	Preserved with minor distortion	Articulated elements; complete
Pes (r)	Preserved with minor distortion	Disarticulated elements; missing distal phalanx and unguals

Phylogenetic Analysis

The phylogenetic position of GP/2E 9266 was accessed using the character-taxon matrix of [14] with 64 taxa (including the new specimen) and 150 discrete characters (supplementary data 1). Parsimony analyses were performed using TNT v. 1.5 Traditional Search algorithm (*Tree Analysis Using New Technology*; [15]), with Wagner trees builds

followed by tree bisection reconnection (TBR), and branch swapping with a hold of 20 and 1,000 replicates, random seed and collapsing trees after the search. Specimen GP/2E 9266 is labelled as *T. navigans* in the analysis.

Computed Tomography (CT) Scanning

The X-ray CT-Scanning was made at Hospital Universitário, Universidade de São Paulo (São Paulo, Brazil), using a Philipp Brilliance 64 medical tomograph. The voxel size of the data is 0.976 mm, with an overlap of 0.33 mm. Resulting tomographic slices were treated and segmented using AVIZO 9.2. The scan stack was upscaled to twice its initial volume using the Resample module and segmented with the brush tool. The generated meshes were smoothed in Blender 2.9 using Laplacian Smooth and Remesh modifiers, and the rendered images were treated in Adobe Photoshop CC 21.1.1.

Institutional abbreviations

AMNH, American Museum of Natural History (New York, USA); CAD, CPCA, Centro de Pesquisas Paleontológicas da Chapada do Araripe (Departamento Nacional de Produção Mineral, Crato, Brazil); GP/2E, Laboratório de Paleontologia Sistemática do Instituto de Geociências da Universidade de São Paulo (São Paulo, Brazil); IMCF, Iwaki Coal and Fossil Museum (Iwaki, Japan); MCT, Museu de Ciências da Terra (Departamento Nacional de Produção Mineral, Rio de Janeiro, Brazil); MN, Museu Nacional (Rio de Janeiro, Brazil); MPSC, Museu de Paleontologia (Santana do Cariri, Brazil); NSM, National Science Museum (Tokyo, Japan); SMNK, Staatliches Museum für Naturkunde (Karlsruhe, Germany); YPM, Yale Peabody Museum of Natural History (New Haven, USA); ZIN, Zoological Institute of the Russian Academy of Sciences (Saint Petersburg, Russia)

Results

Systematic Palaeontology

Pterosauria [16]

Pterodactyloidea [17]

Tapejaridae [18]

Tapejarinae [1]

Tupandactylus [1]

Tupandactylus navigans ([8])

Horizon and locality. Crato Formation, Santana Group, Araripe Basin, NE-Brazil. Lower Cretaceous (Albian). Exact locality undetermined.

Material. GP/2E 9266, an almost complete skeleton with associated soft tissue remains (Table 1; Fig. 1).

Figure 01 – Tupandactylus navigans GP/2E 9266.

Photo of specimen (A); 3D model of specimen (B). Abbreviations: atax, atlas-axis complex; cav, caudal vertebrae; cv, cervical vertebrae; d4, digit four; dc, dentary crest; dov, dorsal vertebrae; f, femur; hu, humerus; il, ilium; isc, ischium; ma, manus; mc, metacarpal; naof, nasoantorbital fenestra; not, notarium; p, pubis; pe, pes; pmc, premaxillary crest; pt, pteroid; rad, radius; sac, sacral vertebrae; sc, scapulocoracoid; spmp, supra-premaxilar bony process; st, sternum; tar, tarsals; tf, tibiofibula; ul, ulna. Scale bar = 50 mm.

Revised diagnosis. *Tupandactylus navigans* can be distinguished from other tapejarid pterosaurs by 1) the autapomorphies from [8]: premaxillomaxilla concave anteriorly; a striated premaxillary crest; supra-premaxillary bony process perpendicular to the long axis of the skull; parietal crest short and rounded; 2) the emended diagnosis: anteriorly

deflected expansion of premaxillary crest; deep and blade-shaped dentary crest with subvertical posterior margin; lateral surfaces of cervical vertebrae postzygapophyses with longitudinal grooves. 3) The phylogenetic analysis recovered two autapomorphies: presence of a notarium; humeral length less than 80% of femoral length.

Description and Comparisons

Generalities

The skull (Table 2; Fig. 2-5) is exposed in left lateral profile, revealing most elements of its antorbital portion and parts of the temporal and occipital portions. The skull bears a notably well-preserved soft-tissue crest that considerably extends dorsally but does not prolong caudally beyond the occiput. The palatal (Fig. 2B) and most the occipital regions (Fig. 3) of the skull are covered by sediment and could only be assessed through CT data. The skull articulates with an edentulous lower jaw, which bears a well-pronounced, anteriorly positioned dentary crest. At the anterior part of the premaxillomaxillae and dentaries, remnants of a keratinous rhamphotheca form narrow patches of soft tissue that extend over the bony limits of the rostrum.

Table 2 – *Tupandactylus navigans* GP/2E 9266 cranium measurements.

Bone	Comments	Measurement (mm)
Cranium	Length from tip of premaxilla to squamosal	286.7
Cranium	Height at the quadrates	88.9
Cranium	Maximum height from dorsalmost tip of sagittal crest to ventral margin of the skull	522.3
Rostrum	Length from tip of premaxilla to anterior of nasoantorbital fenestra	74.4

Rostrum	Height of the anteriormost point of the nasoantorbital fenestra	28.9
Rostrum	Height anterior to nasoantorbital fenestra	161.1
Rostrum	Ventral deflection angle relative to ventral margin of the skull	151º
Rostral Value	RV ratio *sensu* Kellner 2010; 2017	2.57
Rostral Index	RI ratio *sensu* Martill & Naish, 2006	0.46
Premaxillomaxilla	Length from tip to jugal	207.9
Supra-premaxillary process	Preserved height	137.4
Sagittal crest	Anterior margin height from dorsal tip of premaxillary crest	342.9
Sagittal crest	Maximum height from dorsalmost tip of sagittal crest to dorsal margin of the skull	470.9
Nasoantorbital fenestra	Anteroposterior length	129.8
Nasoantorbital fenestra	Dorsoventral height	64.6
Jugal (l)	Maxillary process length	40.5
Jugal (l)	Lacrimal process length	31.8
Jugal (l)	Postorbital process length	51.0
Jugal (l)	Inclination angle of lacrimal process relative to ventral margin of the skull	101º
Jugal (l)	Inclination angle of postorbital process relative to ventral margin of the skull	140º

Jugal (l)	Inclination angle of quadrate process relative to ventral margin of the skull	151º
Quadrate (l)	Inclination angle relative to ventral margin of the skull	148º
Mandible	Length from tip of dentary to retroarticular process	229.6
Mandible	Ventral deflection angle relative to dorsal margin of the mandible	168º
Symphysis	Length from tip of dentary to the posterior margin of the symphysis	94.1
Mandible	Rami separation angle	20º
Mandible	Mid-shaft height	16.4
Mandible	Mid-shaft width	4.1
Dentary Crest	Anteroposterior length	103.3
Dentary Crest	Dorsoventral height	87.1
Dentary Crest	DCH/MRH ratio *sensu* Vullo *et al.*, 2012	5.31
Dentary Crest	Angle between posterior margin of dentary crest to rami	91º
Hyoid	Max preserved length	119.3

Virtually the entire vertebral series is present (Table 3; Fig 6-8), including the atlas/axis complex and some caudal elements. Most vertebrae are in anatomical position, being preserved in left lateral view. The caudal series, however, is slightly displaced and it is not clear whether its elements are rotated in their longitudinal axes. The cervical series is well preserved, but the centra of some elements are laterally crushed. On the other hand,

most dorsal vertebrae are strongly weathered, so that their external bone layers are indistinguishable, exposing chunks of trabecular bone. Nine free vertebrae are present anterior to the notarium, with vertebrae I and II fused forming the atlas/axis complex. Five mid-cervical vertebrae are comparatively long anteroposteriorly, in the typical condition displayed by cervical elements of azhdarchoids. Together with the atlas/axis complex, which is partially covered by the skull, these are here regarded as typical cervicals. Cervical vertebrae 7 to 9 share several features with dorsal elements and are here considered as cervicalized dorsal vertebrae. The large size of individual cervicals makes the cervical series comparable in size with the sum of the lengths of the dorsal and sacral series. As preserved, some mid-cervicals and anterior dorsals are partially covered by forelimb elements. For practical reasons, all pre-notarial free elements are considered as cervical vertebrae on the description below. The five anteriormost dorsals constitute the notarium. Besides post-notarial free dorsal vertebrae, five posterior elements of the dorsal series are fused incorporating the synsacrum. A large sternum (Fig. 9) is also preserved as a plate-like bone just below the vertebral column.

Table 3 - *Tupandactylus navigans* GP/2E 9266 axial skeleton measurements.

**Bone**	**Comments**	**Measurement (mm)**
Atlantoaxis	Centrum length	21.7
Atlantoaxis	Dorsoventral height	40.3
Atlantoaxis	Centrum width	12.7
Cervical Vertebra 3	Centrum length	38.2
Cervical Vertebra 3	Dorsoventral height	21.1

Cervical Vertebra 3	Centrum width	27.0
Cervical Vertebra 4	Centrum length	49.9
Cervical Vertebra 4	Dorsoventral height	21.5
Cervical Vertebra 4	Centrum width	16.6
Cervical Vertebra 5	Centrum length	46.7
Cervical Vertebra 5	Dorsoventral height	21.7
Cervical Vertebra 5	Centrum width	15.3
Cervical Vertebra 6	Centrum length	52.0
Cervical Vertebra 6	Dorsoventral height	24.7
Cervical Vertebra 6	Centrum width	10.3
Cervical Vertebra 7	Centrum length	45.4
Cervical Vertebra 7	Dorsoventral height	27.6

Cervical Vertebra 8	Centrum length	40.2
Cervical Vertebra 8	Dorsoventral height	24.8
Cervical Vertebra 9	Centrum length	23.7
Cervical Vertebrae	Cervical vertebrae total length	317.8
Dorsal Vertebrae	Free dorsal vertebrae centrum average length	14.2
Dorsal Vertebrae	Free dorsal vertebrae centrum average width	15.2
Dorsal Vertebrae	Dorsal and sacral vertebrae total length	211.9
Caudal Vertebrae	Caudal vertebrae total length	34.1
Sternum	Anteroposterior length	132.2

Fore and hind limbs (Tables 4; Fig 10-13) are disarticulated from their girdles, but their elements remain in articulation with each other, preserving even complete autopodia. The wingspan is estimated to reach 2.6 m (measured by the length of preserved forelimb and pectoral girdle elements in anatomical position). To account for the fusion of the skeleton (i.e., sacrum, the proximal extensor processes of the first wing phalanges and the fusion of the notarium), GP/2E 9266 is here interpreted as a subadult individual.

Table 4 - *Tupandactylus navigans* GP/2E 9266 appendicular skeleton measurements

**Bone**	**Comments**	**Measurement (mm)**

Scapula	Length	130.5 (l); 132.4 (r)
Scapula	Mid-shaft diameter	8.0 (l); 10.8 (r)
Coracoid	Length	83.5 (l); 86.8 (r)
Coracoid	Mid-shaft diameter	8.5 (l); 5.8 (r)
Humerus	Length	125.5 (l); 131.0 (r)
Humerus	Mid-shaft diameter	15.5 (l); 19.5 (r)
Humerus	Deltopectoral length	22.5 (l); 25.8 (r)
Humerus	Deltopectoral width	39.9 (l); 30.6 (r)
Ulna	Length	186.1 (l); 188.1 (r)
Ulna	Mid-shaft diameter	13.7 (l); 16.6 (r)
Radius	Length	180.6 (l); 184.1 (r)
Radius	Mid-shaft diameter	10.1 (l); 9.42 (r)
Pteroid	Length	97.0 (r)
Metacarpals 1-3	Max length	82.1 (l); 60.8 (r)
Metacarpal 4	Length	183.9 (l); 181.7 (r)
Metacarpal 4	Mid-shaft diameter	18.5 (l); 16.5 (r)
Manual Phalanx 1 (r)	Length	25.6 (d1); 15.7 (d2); 27.6 (d3)
Manual Phalanx 2 (r)	Length	24.6 (d2); 4.6 (d3)
Manual Phalanx 3 (r)	Length	24.4 (d3)
Manual Unguals (r)	Length	27.3 (d1); 24.5 (d2); 26.4 (d3)
Manual Unguals (r)	Max depth	11.2 (d1); 10.1 (d2); 9.3 (d3)
Wing phalanx 1	Length	317.5 (l); 304.0 (r)
Wing phalanx 1	Mid-shaft diameter	10.5 (l); 12.2 (r)
Wing phalanx 2	Length	193.1 (l); 196.1 (r)
Wing phalanx 2	Mid-shaft diameter	7.1 (l); 6.5 (r)

Wing phalanx 3	Length	123.1 (l); 129.9 (r)
Wing phalanx 3	Mid-shaft diameter	4.1 (l); 4.2 (r)
Wing phalanx 4	Length	39.3 (l)
Wing phalanx 4	Mid-shaft diameter	2.6 (l)
Ilium	Preacetabular length	78.3 (l)
Ilium	Postacetabular process length	44.3 (l)
Pubis	Depth	26.2 (l)
Femur	Length	167.9 (l); 163.1 (r)
Femur	Mid-shaft diameter	14.2 (l); 13.6 (r)
Femur	Angle between head and shaft	46º (r)
Tibia	Length	246.2 (l); 248.8 (r)
Tibia	Mid-shaft diameter	11.7 (l); 10.6 (r)
Fibula	Length	74.2 (l); 67.2 (r)
Metatarsals (l)	Length	35.8 (d1); 44.9 (d2); 47.2 (d3); 46.3 (d4)
Metatarsals (l)	Width	2.6 (d1); 2.4 (d2); 2.5 (d3); 3.0 (d4)
Pes Phalanx 1 (l)	Length	19.2 (d1); 6.4 (d2); 8.5 (d3); 9.4 (d4)
Pes Phalanx 2 (l)	Length	16.5 (d2); 3.5 (d3); 5.4 (d4)
Pes Phalanx 3 (l)	Length	14.1 (d3); 11.9 (d4)
Pes Unguals (l)	Length	11.6 (d1); 14.1 (d2); 12.2 (d3); 10.7 (d4)

Pes Unguals (l)	Max depth	4.2 (d1); 4.7 (d2); 5.5 (d3); 5.1 (d4)

Skull

Premaxillomaxilla

It is not possible to distinguish the premaxilla from the maxilla, as there is no visible suture between these elements. This is a common feature in pterosaurs and was previously observed in other tapejarids [9; 18-19]. The premaxillomaxillae are slightly concave anteriorly, forming a sharp, ventrally oriented rostrum, and extending dorsally to form the premaxillary crest. A sharp rostral tip is commonly observed in tapejarine tapejarids (e.g., *Tapejara wellnhoferi*, *Tupandactylus*, *Caiuajara dobruskii*, *Sinopterus dongi* [20], *Europejara olcadesorum* [2], and *Eopteranodon lii* [21]). The Rostral Value of GP/2E 9266 (as measured from the tip of the premaxillomaxillae to the anterior margin of the nasoantorbital fenestrae [22-23]) is 2.57, an intermediate value when compared with Chinese tapejarids (ranging from 1.76 in "*Huaxiapterus jii*" [19] to 4.05 in *Sinopterus lingyuanensis* [6; 24]). The rostral index (RI *sensu* [25]) of GP/2E 9266 is 0.46, differing from those of the holotype (SMNK PAL 2344) and a referred specimen (SMNK PAL 2343) of *T. navigans* (0.65 and 0.6, respectively), due to the premaxillary crest expansion. Low RI values are reported for short rostra, contrary to the condition found in long-jawed pterosaurs, such as azhdarchids (RI values, 4.36 - 7.33). GP/2E 9266 shares with other tapejarines a downturned anterior end of the rostrum, with a slope of 151º (Fig. 2A). A short and ventrally deflected rostrum is synapomorphic of tapejarine tapejarids [1].

Figure 02 – *Tupandactylus navigans* GP/2E 9266 skull.

3D model in left lateral view (A); palatal view (B). Abbreviations: ec, ectopterygoid; fp, frontoparietal; j, jugal; la, lacrimal; m, maxilla; n, nasal; or, orbit; pl, palatine; pm, premaxilla; po, postorbital; pty, pterygoid; q, quadrate; spmp, supra-premaxilllary bony process; sq, squamosal. Scale bars = 50 mm.

The premaxillomaxillae are posteriorly concave, delimitating the roughly semi-circular anterior margin of the nasoantorbital fenestrae. The maxillae extend posteriorly as thin horizontal plates, reaching the jugals below the orbits and forming the ventral margins of the nasoantorbital fenestrae as well as a considerable portion of the palate. Dorsally, the palates are concave, with maxillary posterolateral processes confining the palatal plate in a deep concavity. For a discussion on the participation of the maxillae in the pterosaur palate, see [26-27].

The presence of a prominent premaxillary crest is shared by all tapejarids. In GP/2E 9266, the exposed bone component of the premaxillary crest is triangular, with a sharp dorsal tip, as was previously observed in *T. imperator* and other *T. navigans* specimens, differing from the rounded crest of *T. wellnhoferi* and the expanded blade of *C. dobruskii*. However, CT data revealed that an anteriorly deflected expansion is present at the most dorsal part of the premaxillary main body (Fig. 2 A), resembling an early ontogenetic stage of *C. dobruskii* premaxillary crest [4]. This expansion is covered by sediment and is possibly also present in the holotype (SMNK PAL 2344). As the remaining premaxillomaxilla, the premaxillary crest is perforated by foramina and has thin grooves probably associated with blood vessels.

The slender and tall supra-premaxillary bony process (Fig. 2A) starts at the dorsal tip of the main bone component of the premaxillary crest and extends dorsally, delimitating the anterior margin of the soft tissue crest. This structure is dorsally broken and reaches the top only as a vestigial groove in GP/2E 9266. A similar bony process is present in *T. imperator*. In this later, however the process deflects posteriorly, contrasting with the perpendicular condition in *T. navigans* [8-9].

Nasals

The nasals (Fig. 2A) are triangular-shaped bones that delimit the dorsoposterior margin of the nasoantorbital fenestra. Lateroventrally this bone contacts the lacrimals, and posteriorly the frontals. Their dorsal limits articulate with the posterodorsal processes of the premaxillae, which form the anterodorsal and dorsal margins of the nasoantorbital fenestra. The suture between nasals and premaxillae cannot be established with confidence. In most tapejarids, this suture seems to be reduced or absent on the lateral surface of the skull ([28], but see specimen CPCA 3590, described by [9]).

Lacrimals

The lacrimals (Fig. 2A) are very thin bones that limit the nasoantorbital fenestrae posteriorly and the orbits anteriorly. They are anteriorly perforated by large foramina, encompassed between their lateral processes and their main rami. In this respect, GP/2E 9266 resembles the condition displayed by *C. ybaka* and *S. dongi*, being very different from the highly perforated lacrimals of *T. wellnhoferi*.

Frontoparietals

Frontals and parietals (Fig. 2A) are fused and laterally compressed in GP/2E 9266. The external surface of the left frontoparietal is partially eroded, but its general outline is preserved. Lateral expansions of the frontals contact the postorbitals and expand laterally to delimit the dorsal margins of the orbits and the anterior margins of the supratemporal

fenestrae. The orbits are positioned high in the skull, with their dorsal portion almost at the same level as the nasoantorbital fenestrae. This is also the condition observed in *T. wellnhoferi*, *C. dobruskii* and *T. imperator*, but differs from the low orbits of *C. ybaka* and thalassodromine tapejarids.

Medially, the parietals compose the inner and posterior margins of the supratemporal fenestrae. The frontoparietals deflects posterodorsally to form a short and blunt crest that does not extend posteriorly further than the posterior limits of the squamosals. This differs from the posteriorly elongated frontoparietal crests of *T. wellnhoferi* and, especially, *T. imperator*.

Jugals

As in other tapejarids (i.e., *T. wellnhoferi*, *C. dobruskii* and what was figured for the holotype of *T. navigans* SMNK PAL 2344), the jugal is a tetraradiate bone (Fig. 2A). Its posterior processes participate in both the upper and lower margins of the lower temporal fenestra. The maxillary process of the jugal delimits the posteroventral margin of the nasoantorbital fenestra. The lacrimal process is thin and slightly deflected posteriorly, forming and angle of little more than 90° with respect to the maxillary ramus.

Quadrates

The quadrates are posteriorly inclined in an angle of 148° (Fig. 2A), falling within the 125°-150° range displayed by other tapejarids (e.g., *Thalassodromeus sethi* ~125°*; T. leonardii* ~130°*; T. wellnhoferi* ~140°*; T. imperator* ~145°*; C. ybaka* ~150°) [28]. This bone is slender medially but forms a deep convex articular surface with the mandible.

Squamosals

The squamosals (Fig. 2A) are broad elements of the temporal region of the skull, forming the dorsal limits of the infratemporal fenestrae and, together with the postorbitals, the

lateral surfaces of the supratemporal fenestrae. Anteriorly, they contact postorbitals over a round and shallow foramen. Squamosal main bodies are broad, with medial flanges that turns into dorsally and ventrally expanded processes. The dorsal process of each squamosal is short and slender when compared to the elongate anteroventrally oriented process. This latter runs parallel to the quadrate, as in *T. wellnhoferi* [29] and "*Tupuxuara" deliradamus* (SMNK PAL 6410, [30]).

Supraoccipital

The supraoccipital (Fig. 3) comprises a considerable part of the occipital area, expanding from the dorsal margin of the foramen magnum to the posteriormost ventral tip of the sagittal crest. Its contact surfaces with squamosals, parietals, opisthotics and exoccipitals are not clear. As in other pterodactyloids, the supraoccipital plate is broad and well developed [29], medially limiting the posttemporal fenestrae.

Figure 03 – *Tupandactylus navigans* GP/2E 9266 skull.

3D model of occipital region of the skull. Abbreviations: bo, basioccipital; bsp, basisphenoid; fm, foramen magnum; eo, exoccipital; j, jugal; p, parietal crest; pcf, postcranial fenestra; ptf, posttemporal fenestra; q, quadrate; so, supraoccipital; sq, squamosal. Scale bar = 50 mm.

Basioccipital

The occipital condyle (Fig. 3) is broad, with a rounded ventral portion and a straight dorsal surface. The basioccipital fuses ventrally with the basisphenoid, forming a plate that encompass most of the ventral area of the occiput. This is also the condition observed in

T. wellnhoferi [29], being a well-developed basisphenoid plate also observed in *C. ybaka* [28].

Palate

The palatal region is covered by sediment and could only be assessed through CT-Scanning (Fig. 2B). Posteriorly, palatines, pterygoids and basipterygoids are preserved, all of them displaying a considerable degree of lateral compression. The maxillae are ventrally fused, forming a palatal plate that extends from the rostrum to level with the anterior limits of the nasoantorbital fenestrae (see [27]). No foramina could be observed on the ventral surface of the maxillary plate, though this is probably a consequence of insufficient resolution of CT data. Similar to what was previously observed in *T. wellnhoferi*, the palate of GP/2E 9266 is concave anteriorly (coinciding with the downslope of the premaxillomaxillae), becoming convex posteriorly. The posterior convexity follows a discrete lateral expansion of the premaxillomaxillae close to the anterior margins of the nasoantorbital fenestrae, and makes GP/2E 9266 posterior palate visible in lateral view. In addition, GP/2E 9266 lacks the deep palatal ridge characteristic of *Tupuxuara leonardii* [27; 31-32]. The anterior margins of the choanae are slightly convex, and the vomers could not be identified.

The palatines apparently form the lateral margins of the choanae and the pterygo-ectopterygoid fenestrae. These bones are slender and anteroposteriorly long. Their contacts with the pterygoids are not discernible, what is common among azhdarchids [27].

Ectopterygoids are well developed in GP/2E 9266 and contact the maxillary bar anteriorly. As in *Pteranodon* and other azhdarchoids (see [27]), the ectopterygoid crosses the pterygoids dorsally, with an angle of ~25º with respect to the anterior ramus of the pterygoid. The contact between ectopterygoid and pterygoid is in the medial process of the latter.

The pterygoids are dorsoventrally thin. The lateral processes of these bones divide the suborbital fenestrae in two, with an elongated and subtriangular pterygo-ectopterygoid fenestra anteriorly and an oval, shorter suborbital fenestra posteriorly. Both fenestrae can be observed laterally in the skull. This same condition was previously reported for *T. leonardii* [27].

Mandible

The edentulous lower jaw is complete (Fig. 4) and preserved still in articulation with the left quadrate. The left mandibular ramus is exposed, while the right mandibular ramus could be only accessed through CT-Scanning. This ramus is ventrally deflected, probably reflecting a pre-burial breakage. The symphysis is located on the first half of the mandible, accounting for 41% of the mandibular length. Tapejarine tapejarids present this element accounting for less than 50% of total mandibular length, with *T. wellnhoferi* bearing the lower value for the symphyseal length relative to the total length of mandible (38%) [33].

Figure 04 – *Tupandactylus navigans* GP/2E 9266 mandible.

3D model in left lateral view (A); dorsal view (B); photograph in left lateral view (C). Abbreviations: d, dentary; dc, dentary crest; glfo, glenoid fossa; hy, hyoids; mc, medial cotyle; rapr, retroarticular process; rmp, rhamphotheca; san, surangular; sym, symphysis. Scale bars = 50 mm.

The symphysis is slightly downturned anteriorly. Close to the deepest region of the dentary crest, the dorsal surface of the symphysis projects dorsally, leveling with the dorsal margins of the mandibular rami. The mandibular rami are laterally compressed,

being the right element broken and deflected downwards, with minor degree of lateral distortion. This allows for the measurement of the angle between both rami (~20º). This angle falls in the range of *T. wellnhoferi* (24º, based on AMNH 24440) and the azhdarchoid *Jidapterus edentus* (20º for the holotype CAD-01; [34]). It is, however, below the ~30º separated rami of *Aymberedactylus cearensis* [33]. The preserved rhamphotheca covers the anterior dorsal concavity of the symphysis, extending ventrally to encase the anterior border of the dentary crest.

Although suture lines between mandibular bones are hard to determine laterally, the dentary was probably the largest element, with the characteristic step-like dorsal margin observed in tapejarines and bearing a deep bony crest. This later extends from the anterior edge of the mandible to almost its midportion, ending in a straight posterior margin that forms an angle of about 90º with the long axis of the lower jaw. The mandibular crest morphology is variable within the Tapejaridae. The ratio between dentary crest height and the mandibular ramus height (see [2] for DCH/MRH) is 5.3 in GP/2E 9266, the highest among tapejarines (2.2 in *S. dongi*; 2.5 in *T. wellnhoferi*, 3 in *T. imperator* and 4 in *E. olcadesorum*). This shows a huge variation even in closely related taxa. In *T. wellnhoferi* this crest is shallower and slightly displaced posteriorly, but still does not surpass the midportion of the jaw (thus smaller in length relative to the condition of GP/2E 9266 and *T. imperator*) [9]. *Sinopterus dongi*, *Huaxiapterus*" *corollatus* [35] and "*Huaxiapterus*" *benxiensis* [36] bear low, blade-like, but longer dentary crests. The dentary crest bears shallow grooves that radiate from its centre to its distal margins. This was first observed for *T. imperator* (CPCA 3590) and may represent deep vascularization of this area or simply be related to the keratinous rhamphotheca anchorage.

The mandibular rami are elongated and shallow, with a height/length ratio of 0.071, a little over *A. cearensis* and under half the ratio of *T. wellnhoferi* (0.142). As in *T.*

imperator, the retroarticular process is short and blunt, differing from *A. cearensis* elongated process.

Hyoid apparatus

The first pair of ossified ceratobranchials is elongated and slender. This element is slightly bowed medially, with an elevated posterior half as in *E. olcadesorum* [2].

Cranial non-ossified tissue

Rhamphotheca

Sheaths of tissue forming a rhamphotheca (Fig. 4C and 5A) cover the anterior and ventral borders of the premaxillomaxillae, as well as the anterior portion of the dentary (extending to the ventral dentary crest). These sheaths have already been figured for *T. navigans* holotype (SMNK PAL 2344), as well as for a referred specimen (SMNK PAL 2343) of this same taxon [8] and were interpreted as keratinous rhamphotheca. This structure, whose outline closely matches that of the rostrum of these specimens, has also been observed in *T. imperator* [9; 37], and inferred for *C. dobruskii* due to presence of abundant lateral and palatal foramina on the external surface of the premaxillomaxillae [4]. Similar foramina are visible in GP/2E 9266 covering the anterior region of the premaxillomaxillae.

Figure 05 – *Tupandactylus navigans* GP/2E 9266 non-ossified tissue. Photograph soft-tissue elements. Abbreviations: apex, apical-most point; lrmp, lower jaw rhamphotheca; sgc, sagittal crest; spmp, supra-premaxillary bony process; trab, trabecular bone; urmp, upper jaw rhamphotheca. Scale bar = 50 mm.

Sagittal crest

The eye-catching prominent soft-tissue crest (Fig. 5B) exceeds five times the skull height at its dorsal most preserved point. The crest is formed by the dorsally and anteriorly striated bones of the premaxillomaxillae, together with a wide portion of soft tissue sustained anteriorly by the elongated, vertically projected supra-premaxillary process. A worth mentioning point is that a well-marked range on the inclination of the anterior most region of this crest is reported for young (~115°) to adult (up to ~90°) individuals in *Caiuajara*, which means that this crest would become steeper as the animal grows [4]. This trend cannot be confirmed for *Tupandactylus* due to the lack of ontogenetic series for the genus. However, if we assume this tendency (to be endorsed by further specimens), it could be suggestive of advanced maturity in individuals with steeper dorsal crests. The fact that all known *T. imperator* specimens have caudally oriented crests while bearing larger skulls than known *T. navigans* (GP/2E 9266, SMNK PAL 2344 and SMNK PAL 2343) may confirm a posteriorly deflected crest as diagnostic for *T. imperator*.

The soft-tissue cranial crest can be divided in two regions: a ventral fibrous crest and a dorsal smooth crest. The sub-parallel vertical pattern of *T. navigans* fibers [8] differs from the one observed in *T. imperator* [9] for the slight anterior orientation (posteriorly oriented in the latter), with no signs of cross-over. The fibrous crest borders the dorsal region of the skull, extending from the basis of the supra-premaxillary crest to the posterior end of frontoparietals. It projects upwards until the transitional region of these striae into the soft-tissue median crest. This differs from what is reported for *T. imperator*, in which fibers contact directly the smooth region of the crest [1; 9].

The smooth crest is convex posteriorly, with some patches of darker perpendicular tissue preserved. Those patches are more apparent at the dorsal region of the crest and describe

a striate pattern. It is worth mentioning that, in GP/2E 9266, this portion of the crest ends dorsally in a concave notch.

Axial skeleton

Cervical vertebrae

Only the posterior part of the atlas/axis complex (Fig. 6 and 7A) is exposed, as these elements are partially covered by the squamosal and occipital bones and were only assessed through CT-Scanning. The axial neural spine has a straight dorsal margin, similar to what is displayed on *Tapejara* (SMNK PAL 1137) and *Tupuxuara* (IMCF 1052). Although the preservation of the atlas-axis complex is rare among the Pterodactyloidea, in taxa like *Pteranodon* (YPM 2440), *Anhanguera piscator* (NSM-PV 19892), *Anhanguera* sp. (AMNH 22555) and *Azhdarcho lancicollis* (ZIN PH 105/44) the axis has a dorsally tapering neural spine terminating in a round surface [38-41]. Atlas and axis are fused (Fig. 7A), with an intervertebral foramen beneath the atlantal neural arch. Apart from the neural spine, the neural arch of GP/2E 9266 axis displays a dorsoventrally deep left postzygapophysis, which is dorsally continuous to a round tubercle. The axial centrum present well developed postexapophyses, as is visible ventral to the left prezygapophysis of cervical vertebra 3.

Figure 06 – *Tupandactylus navigans* GP/2E 9266 cervical vertebrae.

Abbreviations: atax, atlas-axis complex; cv, cervical vertebrae. Scale bars = 10 mm.

Figure 07 – *Tupandactylus navigans* GP/2E 9266 cervical vertebrae.

3D model of atlas/axis complex (A); cervical vertebra 03 (B); cervical vertebra 04 (C); cervical vertebrae series (D); cervical vertebra 05 (E); cervical vertebra 06 (F); cervical

vertebra 07 (G). From upper left to bottom right: left lateral view, anterior view, posterior view, dorsal view and ventral view. Abbreviations: ana, atlantal neural arch; atax, atlas-axis complex; ct, cotyle; cv, cervical vertebrae; ep, epipophysis; fo, pneumatic foramina; nc, neural canal; ns, neural spine; prz, prezygapophysis; pe, postexapophysis; psz, postzygapophysis. Scale bars = 10 mm.

Cervical vertebrae 3-6 (Fig. 6 and 7B-G) have proportionally long centra, albeit not reaching the extreme condition exhibited by azhdarchids. Anteroposterior length of centra increase towards the posterior midcervical series, reaching its maximum in cervical 6. Cervicals 7-9 (Fig. 6 and 7D and G) have shorter central length, but this observation is hindered by the fact that cervical 8 and 9 are poorly preserved. A single small foramen is present on the lateral surface of the midcervical centra. Lateral pneumatic foramina commonly pierce the centra of thalassodromine cervicals [42-44]. As an example, large pneumatic openings of cervicals 2-3 were recognized for a tapejarid specimen (AMNH 22568) by [43]. In contrast, these structures seem to occur more rarely in tapejarines, with a single small foramen being documented for a midcervical of *Tapejara* [5] and two for other tapejarid specimen (MN 4728-V) [43]. Lateral cervical foramina are also rare among the Azhdarchidae, but were reported for cervical 8 of *Azhdarcho lancicollis* [41]. On the other hand, very large foramina in the lateral surface of the centrum are widespread among pteranodontoids (*sensu* [45-46]).

The cervical centra are strongly procoelous, as the cotyles extend posteriorly beyond the postzygapophyses, being easily discernible in lateral aspect. The cotylar region of the centrum of all elements (including the atlas-axis complex) is laterally expanded, with well-developed postexapophyses. In these elements the ventral margin of the centrum is slightly concave. As previously observed, the centra of tapejarine tapejarids cervicals

display concave ventral edges, whereas thalassodromine tapejarids have cervical centra with straight ventral margins [43].

As is typical among the Pterodactyloidea, cervical neural arches are laterally swollen. Despite that the precise spot where the centrum sutures with the neural arch is unclear, a step-like longitudinal ridge roughly separating both structures in lateral aspect is visible. The cervical neural arch is spool-shaped in dorsal view, as the pre- and postzygapophyses consistently diverge laterally from a comparatively constricted midportion. This is a common feature among dsungaripteroids (*sensu* [45]), displayed, for instance, by *Pteranodon* (YPM 2730; [40]), *Anhanguera* (AMNH 22555; [38]), *Tapejara* (SMNK PAL 1137; [5]) and *Tupuxuara* (IMCF 1052). Both pre- and postzygapophyses are prominent and bear wide articular facets. The lateral surfaces of the postzygapophyses display longitudinal grooves starting near the midpoint of the neural arches and terminating in an adjacent region to the articular surfaces. These longitudinal grooves are particularly deep in cervicals 3 and 5 and, to our knowledge, are thus far exclusive for GP/2E 9266. Epipophyses (sometimes referred as postzygapophyseal tubercles, e.g. [47]) are conspicuously present in cervicals 3 to 7. Among the postaxial elements of the cervical series, neural spines are completely exposed in cervicals 3, 4 and 7, with all (excepting the latter) displaying hatched-shaped neural spines with modestly convex dorsal margins. Moreover, in both cervicals 3 and 4 the neural spine has an anterodorsally sloping anterior margin, whereas the posterior one is subvertical. Similar hatched-shaped neural spines were previously reported for indeterminate thalassodromines and tapejarines (e.g. AMNH 24445, AMNH 22568; [43]) and is also present in *Tupuxuara* (IMCF 1052). Neural spine morphology differs sharply in cervical 7, where it assumes an anteroposteriorly long and dorsoventrally deep rectangular shape. In addition, the neural spine of cervical 7 thickens dorsally to form a spine table similar to those displayed by anterior (notarial) dorsal

vertebrae. Cervicals 7, 8 and 9 display anteroposteriorly short centra compared to those of midcervicals, which indicates a transitional morphology between a typical cervical and dorsal elements or, as proposed by [40] for *Pteranodon*, that these are cervicalized dorsal vertebrae.

Dorsal vertebrae

The dorsal series can be divided into three distinct regions: i) five anterior vertebrae with dorsally thickened neural spines, forming a notarium (Fig. 8A, C-E), ii) five free mid dorsals and iii) five synsacral dorsals. Robust transverse processes are visible in dorsals 1, 4 and 5, all three still in association with their corresponding ribs. The neural spines of the three anteriormost dorsal vertebrae are exceptionally well developed, with subvertical anterior and concave posterior margins. They are sequentially in very close association with one another and display transversely thick, spine table-like dorsal ends with the postzygapophysis fused to the prezygapophysis of the subsequent vertebra, which is probably due to ossified tendons associated to the notarial ossification. It is worth mentioning that *C. dobruskii* does not bear a notarium [4], but this structure was reported in an indeterminate tapejarid from the Crato Formation (MN 6588-V).

Figure 08 – *Tupandactylus navigans* GP/2E 9266 dorsal vertebrae and ribs.

3D model of notarium in dorsal view (A); dorsal vertebra 06 in anterior view (B); notarium in anterior view (C); dorsal vertebrae series in left lateral view (D); photograph of notarium (E). Abbreviations: ct, cotyle; not, notarium; ns, neural spine; prz; prezygapophysis; rb, ribs; tpr, transverse process. Scale bar = 20 mm.

Very few relevant features were preserved in the mid free dorsals (Fig. 8B and D). They are anteroposteriorly shorter than cervicals/anterior dorsals, and the better-preserved ones display tall neural spines that differ from those of the first five vertebrae by the absence of a thickened spine table and for having concave anterior and posterior margins. Thoracic vertebrae previously described for *T. wellnhoferi* (SMNK PAL 1137) display either neural spines with subvertical anterior and posterior margins, or convex and concave anterior and posterior margins ([5], Fig. 5).

Synsacral vertebral (Fig. 9A-C and 9D) fusion seems to be similar to that observed in notarial elements, in which the co-ossification of neural spines results from bundles of ossified tendons restricted to the dorsal limits of these structures. Poor preservation prevents an accurate assessment of possible fusion between pre/postzygapophyses and centra of successive synsacral vertebrae, but it is clear that the synsacrum was formed by five individual elements. The Crato Formation tapejarid (MN 6588-V) described by [48] has a similar synsacral configuration as GP/2E 9266.

Figure 09 - *Tupandactylus navigans* GP/2E 9266 sacrum and caudal vertebrae. 3D model of sacrum in left lateral view (A); anterior view (B); dorsal view (C); posterior caudal vertebrae in left lateral view (D); photograph of sacrum (E). Abbreviations: ace, acetabulum; angp, angular process; anilp, anterior ilium process; il, ilium; isc, ischium; p, pubis; sac, sacral vertebrae; vilf, ventral ilium fossa; viscf, ventral ischium fossa. Scale bar = 10 mm.

Caudal vertebrae

Five spindle-shaped caudal vertebrae (Fig. 9D) lie in close association with the posterior elements of the pelvic girdle. They are mainly featureless, not presenting recognizable processes associated to the neural arch. The two presumably anterior elements are robust, with their lengths slightly bigger than their width. The posterior caudals are three times longer than wide (ranging from 5-6.1 mm long x 1.8-2.1 mm wide). It is unlikely that these five elements represent the whole caudal series, but the poor preservation of caudal vertebrae in the azhdarchoid fossil record hinders a proper evaluation of vertebral count for representatives of this clade.

Sternum

The sternum (Fig. 10) is displaced from its anatomical position and exposed in ventral view. Some appendicular elements overlap both lateral margins of this bone, which is also partially covered by rock matrix. The sternal plate reveals to be wide and roughly square-shaped. A large pneumatic foramen pierces the dorsal surface of the sternum, ~~bellow~~ the cristospine. This feature was also observed in *T. wellnhoferi* (SMNK PAL 1137; [5]). Despite being dorsoventrally compressed, the sternum appears to have been considerably convex in its ventral surface. The concave anterolateral margins of the sternal plate converge to contact the cristospine, which is comparatively as long anteroposteriorly as that of *Tupuxuara* (IMCF 1052), and longer than that in *T. wellnhoferi* (SMNK PAL 1137). The posterior margin of the sternal plate appears to be straight, similar to what is displayed by an indeterminate tapejarid (MN 6558-V). *Tapejara wellnhoferi* (SMNK PAL 1137) and *Tupuxuara* (IMCF 1052) present sternal plates with distinctly convex posterior margins.

Figure 10 – *Tupandactylus navigans* GP/2E 9266 sternum.

3D model of sternum in ventral view (A); dorsal view (B); anterior view (C). Abbreviations: cs, cristospine; pf, pneumatic foramina. Scale bar = 20 mm.

Appendicular skeleton and girdles

Scapulocoracoids

Both scapulocoracoids are present in GP/2E 9266, but the left one is better preserved (Fig. 11). It lies in dorsal aspect close to the distal portion of the left hindlimb. The scapulocoracoids are single functional elements formed by the fusion of scapulae and coracoids without any sign of suture lines. A transverse fracture is visible on the proximal part of the coracoidal shaft in the left scapulocoracoid. This could not, however, represent a division line with the scapula, as articulation between scapula and coracoid occur dorsally so that both bones contribute to the glenoid fossa (see [38], Fig. 16). The scapula is considerably longer than the coracoid, what is a common feature among azhdarchoids (e.g. [42; 48]). It expands lateromedially close to the articulation with the coracoid, so that a round supraglenoid process is visible dorsal to the procoracoid in anterior view. Distal to this, the scapular shaft twist along its axis, which makes the scapular blade face laterally. This blade expands progressively towards its distal end, which is broken on the left element, but preserved on the right one, showing a flat articular surface for the notarium. The procoracoid is visible as a well-developed lateral bulge in both scapulocoracoids. The proximal portion of the coracoid expands ventrolaterally to form a deep coracoidal flange as in *Pteranodon* (e.g. YPM 2525; [40]). The indeterminate Crato Formation tapejarid (MN 6588-V) also presents a ventrolateral coracoidal flange, posterior to which a modest tubercle is visible as in GP/2E 9266. Thalassodromine tapejarids present much deeper coracoidal tubercles when compared with MN 6588-V,

what can also be observed in other specimens (e.g. AMNH 22567 and MN 6566-V) [42; 44]. The glenoid fossa is deep and lies between the developed scapular and coracoidal tubercles.

Figure 11 – *Tupandactylus navigans* GP/2E 9266 left scapulocoracoid.
3D model of left scapulocoracoid in dorsal view (A); ventral view (B). Abbreviations: cor, coracoid; crpr; coracoid process; glfo, glenoid fossa; sca, scapula; scpr, scapula process; sglpr, supraglenoid process; tu, coracoid tuberculum. Scale bar = 20 mm.

Humerus

The left humerus is exposed in dorsal view and dorsoventrally flattened, while the right element is ~~preserved~~ in anterior view, with its proximal portion still covered by rock matrix and by the left forelimb phalanges. The left humerus is divided in two slabs and is near complete, lacking some of its distal portion, while the right humerus (Fig. 12) is complete and relatively small when compared to what is observed in other azhdarchoids (hu/fe ~ 0.75) [49-50].

Figure 12 – *Tupandactylus navigans* GP/2E 9266 right humerus.
3D model of right humerus in dorsal view (A); ventral view (B); medial view (C); proximal view (D). Abbreviations: dpc, deltopectoral crest; ect, ectepicondyle; ent, entepicondyle; pf, pneumatic foramen. Scale bar = 10 mm.

Both deltopectoral crests remain covered by sediment and were also assessed through CT data. The crest is well developed and forms ~90º with the humeral shaft. The left deltopectoral crest is anteroposteriorly compressed and the right one is complete and unflatten, being twice as long as it is tall. This structure is slightly curved anteroventrally, as observed in *T. wellnhoferi* [5]. The right humeral head is broad and posteroventrally oriented, contrarily to the dorsal oriented caput of *T. wellnhoferi* [5]. A large subcircular pneumatic foramen is visible beneath the humeral head, on the dorsal margin of the bone, and another foramen is located ventrally, beneath the anterodorsal margin. The presence of both dorsal and ventral pneumatic foramina was observed in ornithocheiroids and *T. wellnhoferi*, but not in *S. dongi* and, thus far, other azhdarchoids [5; 35] or pteranodontids [51].

The humeral shaft is straight and constricted medially, broadening to twice its width at its distal end, with an almost flat radioulnar articular surface. Its medial surface is not as anteroposteriorly compressed as it is in *T. wellnhoferi*, being similar to what is displayed by *C. dobruskii*, and relatively thinner than the condition in earlier pterodactyloids [4-5; 42; 52].

The entepicondyle expands distally and posteriorly, forming the wider dorsodistal margin of the humerus. This expansion is about as broad as the ectepicondyle and not as strongly projected as in pteranodontids [51]. The ectepicondyle is slightly expanded anteriorly as is seen in other azhdarchids [53].

Distally, the ventral surface of the right humerus is flattened, while the dorsal surface is rounded, what gives a D-shaped aspect to the bone cross section. This pattern is characteristic of azhdarchoids, differing from the subtriangular-shaped condition of ornithocheirids [45; 51]. The radial condyle is wider and more spherical than the ulnar

condyle. Both condyles are separated by a deep pneumatic foramen medially located at the distal surface of the humerus.

Radius and Ulna

The left radius and ulna are exposed in ventral view, with the mid/proximal portion of the ulna lying under the left humerus, while its mid/distal portion is partially covered by the right femur and tibia, and both are medially separated in different slabs. The right radius is preserved in anterior view, while the right ulna has its dorsal surface exposed. The ulna is slightly longer than the radius, both being longer than the humerus by 48% and 44%, respectively. Both ulna/humerus and radius/humerus length ratio are similar to *S. dongi* (49% and 42% longer than the humerus, respectively) [20].

Both radius and ulna are straight and gracile. The radius has over half the diameter of the ulna (0.60 of the ulnar shaft diameter). This characteristic is shared with other tapejarids whose radius are slightly over half of the ulnar width (0.57 for *C. dobruskii* CP.V 869, 0.69 for *Tupuxuara leonardii* IMCF 1052) and is further wider in pteranodontids and archaeopterodactyloids (close to 0.7 and over 0.7, respectively) [4; 45]. Although compressed, the ulnae show a gentle, dorsally oriented distal curvature, as found in *T. wellnhoferi* [5; 54].

The ulna has a ventrally expanded prominent articular tubercle at its distal end. In pteranodontids and ornithocheirids this tubercle is medially placed, while ventrally projected in other azhdarchoids and archaeopterodactyloids [51].

Carpus and Metacarpus

Two syncarpal bones, together with the paraxial carpal and the pteroid form the carpal complex. The left carpals (Fig. 13A and D) are exposed in anterior view, while the right carpals lie in posterior view, mostly covered by the right metacarpal IV. The proximal

carpal is concave distally, with its midportion proximo-distally constricted and its medial region prominently expanded both proximally, as an articular facet for ulna, and distally, articulating with the distal carpal. Posteriorly the proximal carpal forms a deep ridge. The distal carpal is a massive, sub-triangular bone that is convex at its proximal part, with an anterior articular tubercle, and almost flat, with a sharp medial condyle, at its distal portion.

Figure 13 – *Tupandactylus navigans* GP/2E 9266 forelimb elements.
3D model of forelimb in dorsal view (A); distal left metacarpus IV in posterior view (B); proximal wing phalanx I in ventral view (C); carpal elements in anterior view (D); left manus (E). Abbreviations: car, carpus; d4, wing phalanxes; dco, dorsal condyle; dsyn, distal syncarpal; etps, extensor tendon process saddle; ft, flexor tubercle; hu, humerus; man, manus; mc, metacarpus; ms, medial sulcus; p, phalanxes; pacar, paraxial carpal; psyn, proximal syncarpal; pte, pteroid; rad, radius; sc, scapulocoracoid; ul, ulna; vco, ventral condyle; vct, ventral cotyle. Scale bar = 50 mm (A); 10 mm (B-E).

The right pteroid (Fig. 13A and D) is very slender and long, reaching over ~0.50 of the ulnar length. Proximally, it has a noticeable posteriorly facing convexity, which gives a curved and slender rod-like shape to this bone, as in other specialized pterodactyloids, such as azhdarchoids, ornithocheirids and pteranodontids [51; 55].

Left metacarpals I-III (Fig. 13E) are exposed, while right metacarpals I-III could only be accessed through CT-Scanning. They do not connect to the carpus, barely reaching the first half of metacarpal IV. This condition has been previously observed in pteranodontids and in other azhdarchoids, such as *S. dongi*, "*H.*" *corollatus* and *Quetzalcoatlus* sp. [20;

35; 56]. Metacarpals I-III are very slender, rod-like bones, with an acute proximal end and a broad articular tubercle on the distal end. The left metacarpal IV (Fig. 12 B) is exposed in ventral view, whereas the right metacarpal IV (Fig. 13A) is in dorsal view and is a long and slender bone. It comprises 0.37 of the “inner wing length”, defined by the humerus, radius/ulna and metacarpal IV, and is relatively shorter than in "*H.*" *corollatus* (0.44) (see [35]). The right metacarpal IV is longer than the humerus (1.46 of humeral length) and has virtually the same length as the ulna. Both proportions closely follow the condition displayed by *T. wellnhoferi* and other tapejarids but are relatively reduced when compared to azhdarchids (2.30 of humeral length in *Quetzalcoatlus* sp.). It is 0.57 of the first wing phalanx length. Ornithocheirids have the metacarpal IV measuring ~0.40 of the first wing phalanx, while apparently all known tapejarids are ~0.60 [57]. The right metacarpal IV is proximally broad, constricting to almost half its width distally. The anteroproximal end is sharp, with a concave cotyle forming the anterior articular surface of the metacarpal with the distal carpus. The proximal posterior end is rounded and forms the articular condyle. The distal end of the metacarpal IV is convex, with a steep anterior articular surface.

Manual Digits

All digits of the left manus are present and complete (Fig. 13A and E), following the phalangeal formula 2-3-4. The unguals preserve a keratinous soft-tissue outline. The abductor tubercle of the ungual of each digit is broad and round. The first phalanx of digit III has the unusual morphology described for *T. wellnhoferi* [5], in which the proximal margin is formed by two proximal condyles separated by a large sulcus. This phalanx is the longest and broadest among the first three digits. This condition differs from that of *T. wellnhoferi*, in which the longest phalanx is that of digit I. Distally, the first phalanx of digits II and III are expanded posteriorly, with a flat articular surface.

The manual unguals are broad, present a strong distal curvature and have over twice the length of the pedal unguals. Most other pterodactyloids are similar in this respect, with the exception of *T. wellnhoferi*, whose manual unguals are less than twice as long as pedal unguals. The unguals articulate with the double distal tubercles of the pre-unguals phalanges, with a broad and round flexor tubercle. A deep medial sulcus crosses the unguals from the distal tip to the proximal end, as noted in other tapejarids (e.g. SMNK PAL 1137).

The left first wing phalanx is partially broken at its proximal end, whereas the right one (Fig. 13A and C) is complete and was preserved with its dorsal side facing upwards. The right first wing phalanx is 2.5 the length of humerus, as in other tapejarids (Eck *et al.*, 2011). Medially, the shaft bows slightly and is anteroposteriorly constricted, with a small round expansion at its distal end. A small pneumatic foramen lies beneath the dorsal cotyle, at the anterior-proximal end, as in several other pterodactyloids [5; 51; 58]. The prominent extensor tendon process forms a posteriorly oriented hook and is totally fused to the proximal region of the bone.

The second (Fig. 13A) and third wing phalanges (Fig. 13A) bear proximal and distal articular expansions as those of the first wing phalanx. The second wing phalanx is 0.61 of the first wing phalanx length, with virtually the same length of the ulna. In other tapejarids such as *T. wellnhoferi* and *C. dobruskii*, the second and first wing phalanx ratios are higher (second wing phalanx length/first wing phalanx length = 0.84 and 0.91, respectively), but much smaller in azhdarchids (e.g. 0.50 in *Quetzalcoatlus* sp.). When comparing the third wing phalanx with the first one, the ratio moves even wider apart. The right third phalanx is ventrodorsally compressed to a third of the anteroposterior width and is 0.38 the length of the first wing phalanx (0.65 in *T. wellnhoferi*, 0.96 in *C.*

dobruskii and 0.29 in *Quetzalcoatlus* sp.). The fourth wing phalanx (Fig. 12A) is the shortest, being mainly featureless.

Pelvis

The pelvis (Fig. 9A-C; 9E) is exposed in left lateral view and is almost complete, lacking only the prepubis and some minor parts of the postacetabular process of the pubis. No suture lines between individual elements can be discernible, which is sometimes used as a proxy for ontogenetic maturity [59-62]. The preacetabular process of the ilium is very long and machete-shaped, displaying in lateral view its wider surface that faces dorsally in most pterosaurs (e.g. [60-61]). As such, it is likely that lateral compression caused this projection to rotate laterally along its main axis, so that GP/2E 9266 exposes the dorsal surface of the process in lateral view. The margin directed dorsally (presumably the medial margin of the preacetabular process) is slightly concave, running almost parallel to the main axis of the sacral vertebral series. In contrast, the margin directed ventrally (assumed to be the lateral one) is shaped as a moderately strong convexity. If the preacetabular process of the ilium indeed rotated laterally, it strongly resembles that of *T. wellnhoferi* (SMNK PAL 1137; [5]). Both margins of this process converge anteriorly to form a pointed tip. Its ventral margin thickens into a crest that dorsally limits a concave anterior flange of the pubis where the suture line between the pubis and the ilium would probably be located.

The ilium expands posteriorly to form a fan-shaped postacetabular process with a constricted base and a very convex posterodorsal margin that terminates in a thick, anteriorly expanded ridge. Although a fan-shaped postacetabular process with a constricted shaft is also present in some isolate archaeopterodactyloids (e.g. *Pterodactylus*), the condition displayed by GP/2E 9266 appears to be restricted to azhdarchoids and is present, for instance, in *T. wellnhoferi* (SMNK PAL 1137; [5]) and

in an indeterminate tapejarid (MN 6588-V). The acetabulum is partially filled with rock matrix and comprises a wide oval aperture anterodorsally delimited by a moderately thick ridge. The pubis is a short, blunt and anteroventrally directed element that connects posteriorly with the ischium to form the ischiopubic plate. This latter appears not to have reached its maximum development as its ventral margin is concave and the pubis stands out as an individualized element. As pointed out by [62-63], the ischiopubic plate formation occurs in late ontogeny. The obturator foramen is wide and oval, with its long axis running anteroposteriorly. It opens ventrally to the acetabulum, in a similar position to that displayed by the specimen MN 6599-V.

Femur

Both femora are present, with the left element exposed in posterior view, and the right one (Fig. 14A-C) in dorsal view. These elements are ~1.28 the length of humerus, and although this ratio is featured by most azhdarchoids, the femora are almost the same size as humerus (1.01) in *C. dobruskii* and is disparate in azhdarchids such as *Zhejiangopterus linhaiensis* (1.48) [4; 35; 64-65].

Figure 14 – *Tupandactylus navigans* GP/2E 9266 hindlimb elements.

3D model of right femur in anterior view (A); medial view (B); proximal region in medio-posterior view (C); left tibia and fibula in anterior view (D); left pes (E). Abbreviations: ast, astragalus; cal, calcaneum; dta, distal tarsus; fb, fibula; gtr, great trochanter; h, femoral head; ms, medial sulcus; mt, metatarsus; p, phalanxes; pf, pneumatic foramen; tb, tibia. Scale bar = 20 mm (A-D); 10 mm (E).

The femoral head (assessed by CT data) forms a 134º angle with the shaft, a similar condition to the majority of pterosaurs excepting pteranodontids and ornithocheirids [64; 66-67]. The femoral neck is constricted medially, and the round caput is prominent, as it is in other azhdarchoids and pteranodontids [51]. The great trochanter is subtriangular shaped and bears a proximal expansion, but lacks the anterior expansion found in other azhdarchoids such as *Quetzalcoatlus* and *T. leonardii*. The lateral trochanter expands distally, forming a round convexity near the fourth trochanter. A small pneumatic foramen lies between the greater trochanter and the femoral neck.

The shaft bows posteriorly on lateral view but is straight at its anterior and posterior views. As noted for other azhdarchoids, the fibular condyle expands posterodorsally and comprises most of the distal expansion of femur. The distal intercondylar fossa is prominent as in *T. wellnhoferi* and thalassodromine tapejarids [5; 42].

Tibia and Fibula

Both tibiae are exposed in anterior view (Fig. 14D). They are gracile bones, being approximately 50% longer than the femur (1.49 the femoral length). Elongated tibiae are present in other azhdarchoids, but not to the same extent as in dsungaripterids such as *Noripterus complicidens* (GIN125/1010; over 1.7) [68].

The tibia is proximally twice as broad as it is distally, with a very prominent proximal tubercle that is absent in thalassodromine tapejarids. The articular surface of tibia is flattened as in other tapejarids, what differs from the rounded surface of ornithocheirids [42]. The left fibula is reduced to less half the length of the tibia (0.39), a ratio just a little below other tapejarine tapejarids (e.g. 0.49 in *T. wellnhoferi*), but proportionally longer than in azhdarchids and dsungaripterids (0.16 in *Quetzalcoatlus* and 0.22 in *N. complicidens*). There is a small round expansion at the proximal end of the fibulae where two distal condyles are separated by a deep fossa.

Tarsals and metatarsals

The proximal tarsals (Fig. 14E) are formed by the calcaneum and astragalus. The former is flattened dorsoventrally, with an overall rectangular shape. The astragalus is concave posteriorly and convex anteriorly, being half-moon shaped, as in other tapejarids (such as *S. atavismus* and *T. wellnhoferi*; [5-6]). Two distal tarsals are present, the left one being longer (10.2 mm) than the right one (6.2 mm). The left distal tarsal is rectangular shaped, whereas the right distal tarsal is sub-triangular. All metatarsals are similar in length and width.

Pes

As in all later-diverging pterodactyloids, there are only four pedal digits. Right metatarsals preserve their distal portion, with the fourth metatarsal being relatively shorter than the first three ones. The phalanges are thin and elongated, with phalangeal formula "2-3-3-4-0" (Fig. 13E). Pedal unguals are shorter (10.7-14.1 mm long) than manual unguals (24.5-27.3 mm long), but present the same mediolateral sulcus, broad and rounded flexor tubercles and a distal ventral curvature.

Phylogenetic analysis

The phylogenetic analysis recovered 132 MTPs of 371 steps each, CI = 0.613 and RI = 0.867 (Fig. 15). The results show the same recovered topology of [14]. GP/2E 9266 was recovered as a member of the clade Tapejarinae, along a polytomy with *T. wellnhoferi*, *T. imperator*, *E. olcadesorum*, and *C. dobruskii*. This clade is supported by one unambiguous synapomorphy [deep, broad in lateral view ossified dentary sagittal crest (84)]. *Tupandactylus navigans* possesses two autapomorphies [presence of a notarium (113); humeral length less than 80% of femoral length (127)].

Figure 15 – Phylogenetic analysis using the character matrix of [14].

Strict-consensus tree recovered from the phylogenetic analysis. Bremer support (> 1) is found over the nodes, and bootstrap (> 50) under the nodes.

Discussion

Ontogeny of GP/2E 9266 and new information on *Tupandactylus navigans* anatomy

Specimen GP/2E 9266 (Fig. 16) is the most complete Brazilian tapejarid described thus far. Although *Tapejara wellnhoferi* and *Caiuajara dobruskii* are known from several individuals, no single specimen preserved the skeleton to the same degree as GP/2E 9266. The new material presents all the diagnostic characteristics of *T. navigans*, namely a striated premaxillary crest, a perpendicular supra premaxillary bony process, and a short parietal crest [8]. In addition, previously unrecorded diagnostic features distinguish *T. navigans* from other tapejarine pterosaurs, such as pterygoids laterally visible (also observed in *T. leonardii* and *T. sethi*), deep, blade-shaped dentary crest (dentary crest height/mandibular ramus height (DCH/MRH) ratio = 5.3) with a subvertical posterior margin, cervical postzygapophyses displaying lateral longitudinal, presence of a notarium and manual unguals over twice the size of pedal unguals. This is also the first time some features are reported for the Tapejarinae, such as the fusion of the atlas-axis complex (which is probably ontogenetically controlled), presence of a synsacral supraneural plate (absent in *V. daisymorrisae*), and ~~metacarpal IV articulating with carpal, with~~ metacarpals I-III only reaching the first half of metacarpal IV. The presence of both dorsal and ventral foramina in the humeri of GP/2E 9266 is also worth noting. This feature was described as autapomorphic for *T. wellnhoferi* [5] but can be a synapomorphy of Tapejarinae.

Figure 16 – Skeletal reconstruction of *Tupandactylus navigans* specimen GP/2E 9266.

Skeletal reconstruction recovered with the segmentation of all bony elements of specimen GP/2E 9266. Scale bar = 50 mm.

The fusion of the premaxillomaxilla is reported even for immature pterodactyloid specimens, demonstrating that it occurred early in ontogeny [39]. Some other osteological features, however, apparently suggest an advanced ontogenetic stage for GP/2E 9266, such as the fusion of the extensor tendon process [58; 62; 69-71] and anterior dorsal vertebrae fused into a notarium. Notarium development of the new specimen fits state SN4 of [72]. According to these authors, stage SN4 is the peak of notarium fusion in azhdarchoids, although new specimens with preserved notaria might show further development of this structure. In GP/2E 9266, the unfused tibiotarsus is the single skeletal feature arguing for a not fully mature ontogenetic stage [73] but, as tapejarid ontogeny is not fully understood, it is still unclear how *T. navigans* fits ontogenetic models created based on distinct pterosaur taxa. As such, the integration of all those proxies for individual maturity indicates that GP/2E 9266 was almost fully developed at the time of death.

T. navigans vs. T. imperator

When first described, *T. navigans* was differed from *T. imperator* based on the perpendicularly oriented sagittal crest and the absence of a posterior expansion of the parietal crest [8]. The new material furthers differentiates the two species, and this cannot be explained by taphonomy or ontogeny: specimens from both show similar preservation and presumably belonged to mature individuals. Here we show that, in addition to the features above, *T. navigans* and *T. imperator* also differ in characters such as the dentary

crest, proportionally larger in *T. navigans*. Additionally, in *T. imperator* (CPCA 3590) the dentary crest is steeper anteriorly, whereas in *T. navigans* (GP/2E 9266) the posterior margin of the crest is steeper. Known specimens of *T. navigans* are smaller than those of *T. imperator*, and the skull proportions vary between both taxa (length-height ratio of 3.2 in GP/2E 9266 and 3.6 for *T. imperator*). It is worth mentioning that for the holotype SMNK PAL 2344, whose skull is relatively longer than GP/2E 9266, the length-height ratio is 2.5 (as noted in [9]).

These differences, however, do not eliminate the possibility of sexual dimorphism. Both the sagittal and dentary crests might have worked as mating displays, what is arguable for pterosaur species evidencing strong allometric growth or definite crest-related sexual dimorphism (e.g. [4; 69; 74-75]). If *T. navigans* and *T. imperator* are indeed two independent morphotypes of a single, sexually dimorphic species, mutual sexual selection is not discarded. This hypothesis, however, may only be tested through the discovery of additional specimens of both *T. navigans* and *T. imperator*.

Non-ossified tissue

A peculiarity of rhamphotheca in GP/2E 9266 is the gap it forms between the premaxillary and dentaryx. This gap prevents total occlusion of *T. navigans* jaws, what could be related to a particular feeding strategy. The presence of a presumably keratinous rhamphotheca covering the anterior rostrum of edentulous pterosaurs was widely reported. Analogously to birds, this structure has been associated with energy storage and absorption of transmitted loads to the bone during the bite, what may be related to the lack of teeth [76]. In pterosaurs such as *T. navigans*, the rhamphotheca could have been a hook-like structure operating as a peg to catch or to manipulate small food items [76].

The soft-tissue sagittal crest of GP/2E 9266 (Fig. 5) is almost completely preserved, with only the apical region either missing or embedded in the sediment. Therefore, GP/2E 9266 possesses the most complete soft-tissue sagittal crest among tapejarines. The convex posterior margin of the crest displayed by the new specimen was suggested for *T. imperator* (CPCA 3590, [9]) and for *T. navigans* holotype [8]. As in CPCA 3590, differential color patterns in the soft-tissue crest may be related to oxidation. A thorough study of soft-tissue preservation in GP/2E 9266 applying UV-light and SEM is already underway.

Implications to flight and terrestrial foraging capabilities

The presence of a notarium in GP/2E 9266 is a novel feature for tapejarines. This structure was not observed in *C. dobruskii*, a pterosaur species from which a presumably complete ontogenetic series is known. The fusion of the first dorsal vertebrae into a notarium allowed for bending and torsion resistance, which helped pterosaur active flight [50]. Also, GP/2E 9266 displays a deltopectoral crest forming a broad surface for muscle anchorage, which probably allowed burst flight. The long fourth metacarpal and slender hindlimbs would have made a standing quadrupedal takeoff feasible [50; 77]. Ultimately, wing phalanges length compared to the inner-wing length in GP/2E 9266 is comparatively short. As such, the characteristic forelimb hypertrophy of specialized pterodactyloids is only moderately present in *T. navigans* [38; 57; 78]. In addition to the long cervical series, these features may indicate a terrestrial stalking lifestyle, similar to what was suggested for azhdarchids [65; 79], albeit also arguing for a seemingly good takeoff capability. The influence of the sagittal crest in both flight and terrestrial capabilities, however, requires further studies. [8] argued that, to have a functional aerodynamic crest, *T. navigans* should have had a short neck or tendon locks on its cervical vertebrae. Neither are observed in GP/2E 9266, in which the cervical series comprises over 55% of the total axial length

(317 mm x 564 mm of the total length), and no ossified tendons are visible in pre-notarial vertebrae. This could indicate that the aberrant crest may have restricted *T. navigans* to short-distance flights, such as to flee from predators.

MN 6588-V as Tupandactylus

[48] assigned specimen MN 6588-V to Tapejaridae mainly based on the presence of a tuberculum at the ventroposterior margin of the coracoid. The overall morphology of MN 6588-V closely resembles GP/2E 9266 in both proportion and shape. MN 6588-V differs from GP/2E 9266, however, in two main aspects: the number of fused notarial vertebra and the number of vertebrae in both dorsal and sacral series. MN 6588-V notarium is composed by the first four dorsal vertebrae, whereas in GP/2E 9266 it is composed of five elements. This difference, however, may be related to ontogeny, with MN 6588-V representing an earlier stage of notarial development (SN3 of [72]). MN 6588-V dorsal and sacral vertebral counts are 11 and 7, respectively, against 10 and 5, respectively, in GP/2E 9266. The preserved axial series is also relatively longer in MN 6588-V (dorsal series length = 178.5 mm, against 134.2 in GP/2E 9266). These features argue for a *Tupandactylus* affinity, but due to the abovementioned differences, this specimen may represent *T. imperator* rather than *T. navigans*. If MN 6588-V is indeed a *T. imperator* (what could only be tested through the description of further specimens), the hypothesis of *T. imperator* and *T. navigans* being separate taxa would strengthen.

Conclusion

The specimen we describe is the most complete articulated tapejarid skeleton thus far recovered in Brazil. The premaxillary crest shape, dentary crest proportions and shape, as well as axial and appendicular skeletal anatomy (including the presence of a notarium) are novel features observed in GP/2E 9266, and an emended diagnosis is here proposed

for *Tupandactylus navigans*. Dentary crest morphology differs from all other tapejarids and further strengthens *T. navigans* as distinct from *Tupandactylus imperator*. This work is, however, inconclusive with regard to sexual dimorphism as a hypothesis to explain similarities between *T. navigans* and *T. imperator*. Specimen MN 6588-V is regarded here as *Tupandactylus* sp., despite being distinct from *T. navigans* in some minor characters.

Axial and limb proportions of the new specimen are indicative of a terrestrial stalking habit for *T. navigans*, but the influence of the sagittal crest in the ecology of *T. navigans* is still unresolved. The new specimen considerably improves our understanding of tapejarid anatomy, taxonomy and ecomorphology.

Acknowledgements

The authors would like to thank Ivone Cardoso Gonzales (IGc/USP) for the access of the material. We want to thank Beatriz Verdasca Aceto (MAE/USP) and Antonio Carlos Matioli (Hospital Universitário/USP), for the tomography of the material. We also want to thank Anna Carolina Dias de Almeida (IB/USP) for photos of the material and overall help during the development of the present work. The authors would like to thank Willi Hennig Society for the gratuity of TNT software. VB want to thank Thermo Fisher Scientific for the student license to Avizo software. For allowing access to relevant fossil collections, FLP is indebted to Mark Norell and Carl Mehling (AMNH); Álamo Saraiva and João Kerensky (MPSC); Oliver Rauhut and Markus Moser (SNSB-BSPG); Alexander Kellner and Helder Silva (MN); Eberhard Frey (SMNK); Rainer Schoch (SMNS); Sandra Chapman and Lorna Steel (NHMUK); Dan Pemberton and Matt Riley (CAMSM).

References

1. Kellner AWA, Campos DA. Short note on the ingroup relationships of the Tapejaridae (Pterosauria, Pterodactyloidea). *Boletim do Museu Nacional: Geologia*. 2007; 75, 1–14.

2. Vullo R, Marugán-Lobón J, Kellner AWA, Buscalioni AD, Gomez B, *et al.* A new crested pterosaur from the Early Cretaceous of Spain: the first European tapejarid (Pterodactyloidea: Azhdarchoidea). *PloS one*. 2012; 7, e38900.

3. Martill DM, Smith R, Unwin DM, Kao A, McPhee J, Ibrahim N. A new tapejarid (Pterosauria, Azhdarchoidea) from the mid-Cretaceous Kem Kem beds of Takmout, southern Morocco. *Cretaceous Research*. 2020; 104424.

4. Manzig PC, Kellner AWA, Weinschütz LC, Fragoso CE, Vega CS, *et al.* Discovery of a Rare Pterosaur Bone Bed in a Cretaceous Desert with Insights on Ontogeny and Behavior of Flying Reptiles. *PloS one*. 2014; 9(8), e100005.

5. Eck K, Elgin RA, Frey E. On the osteology of *Tapejara wellnhoferi* Kellner 1989 and the first occurrence of multiple specimen assemblage from the Santana Formation, Araripe Basin, NE-Brazil. *Swiss Journal of Palaeontology*. 2011; 130, 277–96.

6. Zhang X, Jiang S, Cheng X, Wang X. New Material of Sinopterus (Pterosauria, Tapejaridae) from the Early Cretaceous Jehol Biota of China. *Anais da Academia Brasileira de Ciências*. 2019; 91, suppl.2

7. Campos DA, Kellner AWA. Short note on the first occurrence of Tapejaridae in the Crato Member (Aptian), Santana Formation, Araripe Basin, Northeast Brazil. *Anais da Academia Brasileira de Ciências*. 1997; 69, 83–87.

8. Frey E, Martill DM, Buchy MC. A new crested ornithocheirid from the Lower Cretaceous of northeastern Brazil and the unusual death of an unusual pterosaur. In: Buffetaut E, Mazin JM, editors. *Evolution and palaeobiology of pterosaurs*. Geological Society, London, Special Publication. 2003; 217, 55–63.

9. Pinheiro FL, Fortier DC, Schultz CL, Andrade JAFG, Bantim RAM. New information on the pterosaur *Tupandactylus imperator*, with comments on the relationships of Tapejaridae. *Acta Palaeontologica Polonica*. 2011; 56(3), 567–580.

10. Assine ML. Bacia do Araripe. Boletim de Geociências da PETROBRAS. 2007; 15(2), 371-389.

11. Heimhofer R, Martill D. The sedimentology and depositional environment of the Crato Formation. In: *The Crato fossil beds of Brazil: window into an ancient world*. Cambridge University Press. 2007; 44-63.

12. Heimhofer U, Ariztegui D, Lenniger M, Hesselbo SP, Martill DM, Rios- Netto AM. Deciphering the depositional environment of the laminated Crato fossil beds (Early Cretaceous, Araripe Basin, North- eastern Brazil). *Sedimentology*. 2010; 57(2), 677-694.

13. Neumann VH, Cabrera L, Mabesoone JM, Valença LMM, Silva AL. Ambiente sedimentar e facies da seqüência lacustre aptiana-albiana da bacia do Araripe, NE do Brasil. *Simpósio Sobre o Cretáceo do Brasil*. 2002; 6, 37-41.

14. Hone DWE, Fitch AJ, Ma F, Xu X. An unusual new genus of istiodactylid pterosaur from China based on a near complete specimen. *Palaeontologia Electronica*. 2020; 23 (1), a09

15. Goloboff PA, Catalano SA. TNT version 1.5, including a full implementation of phylogenetic morphometrics. *Cladistics*. 2016; 32(3), 221-238.

16. Kaup SS. Versuch einer Eintheilung der Saugethiere in 6 Stämme und der Amphibien in 6 Ordnung. *Isis*. 1834; 311–324.

17. Plieninger F. Beiträge zur Kenntnis der Flugsaurier. *Palaeontographica*. 1901; 48, 65–90.

18. Kellner AWA. A new edentate pterosaur of the Lower Cretaceous from the Araripe Basin, Northeast Brazil. *Anais da Academia Brasileira de Ciências*. 1989; 61: 439–446.

19. Lü J, Yuan C. New tapejarid pterosaur from Western Liaoning, China. *Acta Geologica Sinica*. 2005; 79, 453–458.

20. Wang X, Zhou Z. A new pterosaur (Pterodactyloidea, Tapejaridae) from the Early Cretaceous Jiufotang Formation of Western Liaoning, China and its implications for biostratigraphy. *Chinese Science Bulletin*. 2003; 48, 16-23.

21. Lü J, Zhang B. New pterodactyloid pterosaur from the Yixian Formation of Western Liaoning. *Dizhi Lunping (Geological Review)*. 2005; 51(4), 458-462.

22. Kellner AWA. Comments on the Pteranodontotidae (Pterosauria, Pterodactyloidea) with the description of two new species. *Anais da Academia Brasileira de Ciências.* 2010; 82, 1063–1084.

23. Kellner AWA. Rebuttal of Martin-Silverstone *et al.* 2017. Reassessment of *Dawndraco kanzai* Kellner 2010 and reassignment of the type specimen to *Pteranodon sternbergi* Harksen, 1966. *Vertebrate Anatomy Morphology Paleontology.* 2017; 3, 81–89.

24. Lü JC, Teng FF, Sun DY, Shen CZ, Li GQ, Gao X, Liu HF. The toothless pterosaurs from China. *Acta Geologica Sinica.* 2016; 90, 2513-2525.

25. Martill DM, Naish D. Cranial crest development in the azhdarchoid pterosaur *Tupuxuara*, with review of the genus and tapejarid monophyly. *Palaeontology.* 2006; 49, 925–941.

26. Ősi A, Prondvai E, Frey E, Pohl B. New interpretation of the palate of pterosaurs. *The Anatomical Record: Advances in Integrative Anatomy and Evolutionary Biology*. 2010; 293(2), 243-258.

27. Pinheiro FL, Schultz CL. An unusual pterosaur specimen (Pterodactyloidea,? Azhdarchoidea) from the Early Cretaceous Romualdo Formation of Brazil, and the evolution of the pterodactyloid palate. *PloS one*. 2012; 7(11).

28. Kellner AWA. A new unusual tapejarid (Pterosauria, Pterodactyloidea) from the Early Cretaceous Romualdo Formation,AraripeBasin, Brazil. *Earth and Environmental Science Transactions of the Royal Society of Edinburgh.* 2013; 103, 1–14.

29. Kellner AWA. Description of the braincase of two early Cretaceous pterosaurs (Pterodactyloidea) from Brazil. *American Museum Novitates*. 1996; 3175: 1–34.

30. Witton MP. A new species of *Tupuxuara* (Thalassodromidae, Azhdarchoidea) from the Lower Cretaceous Santana Formation of Brazil, with a note on the nomenclature of Thalassodromidae. *Cretaceous Research*. 2009; 30(5), 1293–1300.

31. Kellner AWA, Campos DA. Sobre um novo pterossauro com crista sagital da Bacia do Araripe, Cretáceo Inferior do Nordeste do Brasil. *Anais da Academia Brasileira de Ciências*. 1998; 60, 459–469.

32. Kellner AWA, Campos DA. A new species of Tupuxuara (Pterosauria, Tapejaridae) from the Early Cretaceous of Brazil. *Anais da Academia Brasileira de Ciências.* 1994; 66, 467–473.

33. Pêgas RV, Leal MEdC, Kellner AWA. A Basal Tapejarine (Pterosauria;Pterodactyloidea; Tapejaridae) from the Crato Formation, Early Cretaceous of Brazil. *PloS one.* 2016; 11(9), e0162692.

34. Dong Z, Sun, Y, Wu S. On a new pterosaur from the Lower Cretaceous of Chaoyang Basin, Western Liaoning, China. *Global Geology*. 2003; 22(1), 1–7.

35. Lü J, Jin S, Unwin D, Zhao L, Azuma Y, *et al*. A new species of *Huaxiapterus* (Pterosauria: Pterodactyloidea) from the Lower Cretaceous of western Liaoning, China with comments on the systematics of tapejarid pterosaurs. *Acta Geologica Sinica.* 2006; 80, 315–326.

36. Lü J, Gao Y, Xing L, Li Z, Ji Q. A New Species of *Huaxiapterus* (Pterosauria: Tapejaridae) from the Early Cretaceous of Western Liaoning, China. *Acta Geologica Sinica*. 2007; 81(5), 683-687.

37. Martill DM, Bechly G, Loveridge RF. (eds). *The Crato fossil beds of Brazil: window into an ancient world*. Cambridge University Press, Cambridge, 2007; 625 pp.

38. Wellnhofer P. The skull of *Tapejara wellnhoferi* Kellner (Reptilia, Pterosauria) from the Lower Cretaceous Santana Formation of the Araripe Basin, Northeastern Brazil. *Mitteilungen der Bayerischen Staatssammlung für Paläontologie und Historische Geologie*. 1991; 31, 89-106.

39. Kellner AWA, Tomida Y. Description of a new species of Anhangueridae (Pterodactyloidea) with comments on the pterosaur fauna from the Santana Formation (Aptian–Albian), Northeastern Brazil. *National Science Museum Monographs.* 2000; 17, 1–135.

40. Bennett SC. The osteology and functional morphology of the Late Cretaceous pterosaur Pteranodon part 1 - General description and osteology. *Palaeontographica*. 2001; 260, 1–112.

41. Averianov AO. The osteology of *Azhdarcho lancicollis* Nessov, 1984 (Pterosauria, Azhdarchidae) from the Late Cretaceous of Uzbekistan. Proceedings of the Zoological Institute RAS. 2010; 314, 264–317.

42. Aires A, Kellner AWA, Müller R, Da Silva LR, Pacheco C, Dias-da-Silva S. New postcranial elements of the Thalassodrominae (Pterodactyloidea, Tapejaridae) from the Romualdo Formation (Aptian–Albian), Santana Group, Araripe Basin, Brazil. Palaeontology. 2014; 57.

43. Vila Nova BC, Sayão JM, Langer MC, Kellner AWA. Comments on the cervical vertebrae of the Tapejaridae (Pterosauria, Pterodactyloidea) with description of new specimens. *Historical Biology*. 2015; 27(6), 771-781.

44. Buchmann R, Rodrigues T, Polegario S, Kellner AWA. New information on the postcranial skeleton of the Thalassodrominae (Pterosauria, Pterodactyloidea, Tapejaridae). *Historical Biology*. 2018; 30, p. 1139-1149.

45. Kellner AWA. Pterosaur phylogeny and comments on the evolutionary history of the group. In: E. Buffetaut and J.-M. Mazin (eds.), *Evolution and Palaeobiology of Pterosaurs*. Special Publication 217. Geological Society, London. 2003. pp. 105-137.

46. Buchmann R, Rodrigues T. The evolution of pneumatic foramina in pterosaur vertebrae. *Anais da Academia Brasileira de Ciências*. 2019; 91, supl. 2, p. e20180782.

47. Liu DX, Zhou CF, Wang JQ, Li WG, Wei QW. New data on the cervical morphology of the Chinese tapejarine. *Historical Biology*. 2014; 27(6), 638-645.

48. Sayão JM, Kellner AWA. Novo esqueleto parcial de pterossauro (Pterodactyloidea, Tapejaridae) do Membro Crato (Aptiano), Formação Santana, Bacia do Araripe, Nordeste do Brasil. *Estudos Geológicos*. 2006; 16(2), 16-40.

49. McGowen MR, Padian K, De Sosa MA, Harmon RJ. Description of *Montanazhdarcho minor*, an azhdarchid pterosaur from the Two Medicine Formation (Campanian) of Montana. *PaleoBios*. 2002; 22 (1), 1-9.

50. Witton MP, Habib MB. On the size and flight diversity of giant pterosaurs, the use of birds as pterosaur analogues and comments on pterosaur flightlessness. *PloS one*. 2010; 5(11), e13982.

51. Longrich NR, Martill DM, Andres B. Late Maastrichtian pterosaurs from North Africa and mass extinction of Pterosauria at the Cretaceous-Paleogene boundary. *PLoS Biology*. 2018; 16(3), e200166.

52. Andres B, Clark J, Xu X. The earliest pterodactyloid and the origin of the group. Current Biology. 201424(9), 1011±6.

53. Agnolin FL, Varricchio D. Systematic reinterpretation of *Piksi barbarulna* Varricchio, 2002 from the Two Medicine Formation (Upper Cretaceous) of Western USA (Montana) as a pterosaur rather than a bird. Geodiversitas. 2012; 34(4), 883-894.

54. Elgin RA, Campos HBN. A new specimen of the azhdarchoid pterosaur *Tapejara wellnhoferi*. *Historical Biology*. 2012; 24(6), 586-591.

55. Vidovic SU, Martill DM. *Pterodactylus scolopaciceps* Meyer, 1860 (Pterosauria, Pterodactyloidea) from the Upper Jurassic of Bavaria, Germany: the problem of cryptic pterosaur taxa in early ontogeny. *PloS one*. 2014; 9(10), e110646.

56. Lawson DA. Pterosaur from the latest Cretaceous of West Texas: discovery of the largest flying creature. *Science*. 1975; 187(4180), 947-948.

57. Vila Nova BC, Sayão JM. On wing disparity and morphological variation of the Santana Group pterosaurs, *Historical Biology*. 2012; 24(5), 567-574.

58. Myers TS. A new ornithocheirid pterosaur from the Upper Cretaceous (Cenomanian–Turonian) Eagle Ford Group of Texas. *Journal of Vertebrate Paleontology*. 2010; 30 (1), 280-287.

59. Bennett SC. Year-classes of pterosaurs from the Solnhofen Limestone of Germany: taxonomic and systematic implications. *Journal of Vertebrate Paleontology*. 1996; 16(3), 432-444.

60. Naish D, Simpson M, Dyke G. A new small-bodied azhdarchoid pterosaur from the Lower Cretaceous of England and its implications for pterosaur anatomy, diversity and phylogeny. *PloS one*. 2013; 8 (3), e58451.

61. Hyder ES, Witton MP, Martill DM. Evolution of the pterosaur pelvis. *Acta Palaeontologica Polonica*. 2014; 59(1), 109-125.

62. Kellner AWA. Comments on Triassic pterosaurs with discussion about ontogeny and description of new taxa. *Anais da Academia Brasileira de Ciências.* 2015; 87(2), 669–689.

63. Bennett SC. An arboreal leaping origin of flight and the relationships of pterosaurs. *Journal of Vertebrate Paleontology*. 1995; 15 (Supplement to number 3), 19A.

64. Unwin DM. On the phylogeny and evolutionary history of pterosaurs. *Geological Society, London, Special Publications.* 2003; 217, 139-190.

65. Witton MP, Naish D. A reappraisal of Azhdarchid pterosaur functional morphology and paleoecology. *PloS one.* 2008; 3, e2271.

66. Andres B, Myers TS. Lone star pterosaurs. Earth and Environmental Science Transactions of the Royal Society of Edinburgh. 2013; 103, 383-398.

67. McLain MA, Bakker RT. Pterosaur material from the uppermost Jurassic of the uppermost Morrison Formation, Breakfast Bench Facies, Como Bluff, Wyoming, including a pterosaur with pneumatized femora. *Geological Society, London, Special Publications*. 2018; 455(1), 105-124.

68. Lü J, Azuma Y, Dong Z, Barsbold R, Kobayashi Y, Lee YN. New material of dsungaripterid pterosaur (Pterosauria: Pterodactyloidea) from western Mongolia and its palaeoecological implications. *Geological Magazine.* 2009; 146(5), 690-700.

69. Bennett SC. The ontogeny of *Pteranodon* and other pterosaurs. *Paleobiology*. **1993;** 19(01), 92±106.

70. Frey E, Martill DM. Soft tissue preservation in a specimen of Pterodactylus kochi (Wagner) from the Upper Jurassic of Germany. *Neues Jahrbuch für Geologie und Paläontologie-Abhandlungen*. 1998; 421-441.

71. Kellner AWA. Pterosaurs: natural history, evolution, anatomy. *The Quarterly Review of Biology*. 2015; 90, 206–207.

72. Aires AS, Reichert LM, Müller RT, Pinheiro FL, & Andrade MB. Development and evolution of the notarium in Pterosauria. Journal of Anatomy. 2020.

73. Kellner AWA. The ankle structure of two pterodactyloid pterosaurs from the Santana Formation (Lower Cretaceous), Brazil. *Bulletin of the American Museum of Natural History*. 2004; (285), 25-35.

74. Wang X, Kellner AWA, Jiang S, Wang Q, Ma Y, Paidoula Y, Cheng X, Rodrigues T, Meng X, Zhang J, Li N, Zhou Z. Sexually dimorphic tridimensionally preserved pterosaurs and their eggs from China. *Current Biology*. 2014; 24, 1323–1330.

75. Pinheiro FL, Rodrigues T. *Anhanguera* taxonomy revisited: is our understanding of Santana Group pterosaur diversity biased by poor biological and stratigraphic control? *PeerJ*. 2017; 5, e3285

76. Fastnacht M. Jaw mechanics of the pterosaur skull construction and the evolution of toothlessness. Unpublished PhD Thesis, *Mainz.* 2005; 228.

77. Habib MB. Comparative evidence for quadrupedal launch in pterosaurs. *Zitteliana.* 2008; 159-166.

78. Dyke GJ, Nudds RL, Rayner JMV. Limb disparity and wing shape in pterosaurs. *Journal of Evolutionary Biology*. 2006; 19, 1339–1342.

79. Witton MP, Naish D. Azhdarchid pterosaurs: water-trawling pelican mimics or “terrestrial stalkers”?. *Acta Palaeontologica Polonica.* 2013; 60(3), 651-661.

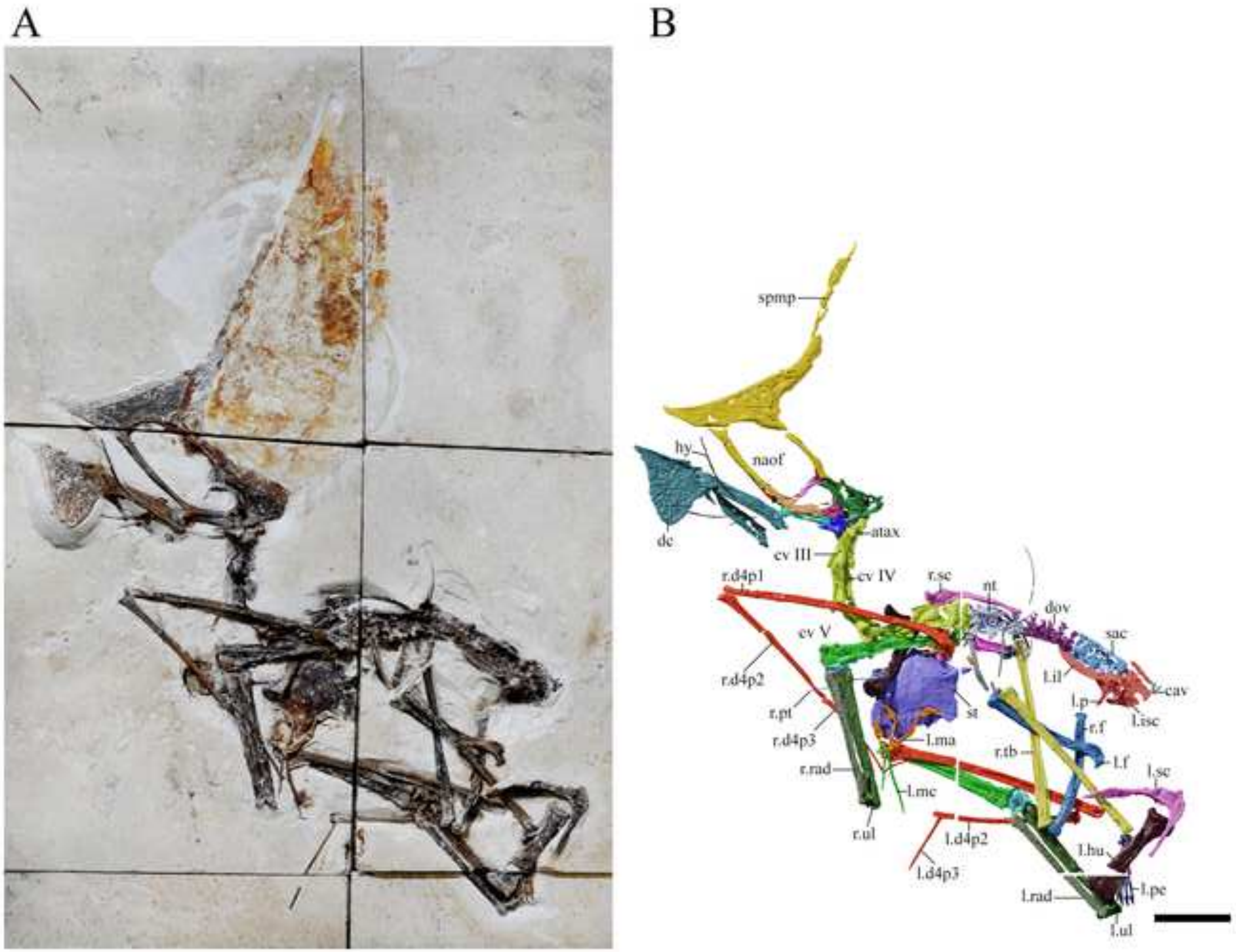
A
B
spmp
hy
naof
dc
atax
cv III
cv IV
r.d4p1
r.sc
nt
dov
cv V
sac
r.d4p2
l.il
cav
st
l.p
l.isc
r.pt
r.f
r.d4p3
l.ma
r.tb
l.f
r.rad
l.sc
l.mc
r.ul
l.d4p2
l.hu
l.d4p3
l.pe
l.rad
l.ul

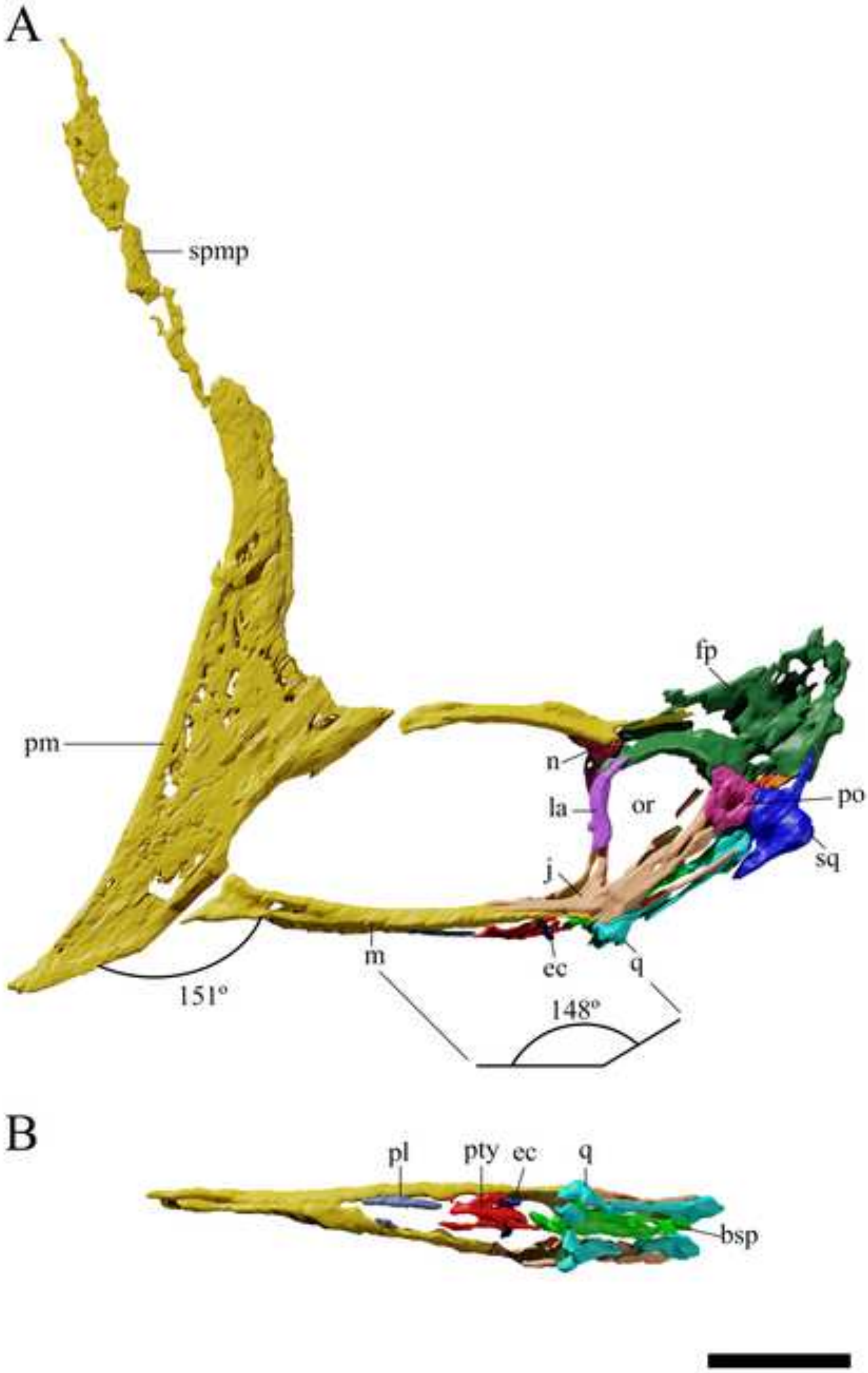
A
spmp
fp
pm
n
la
or
po
sq
j
m
ec
q
151°
148°
B
pl
pty
ec
q
bsp

Figure 3

Click here to access/download;Figure;Figure 3.tif

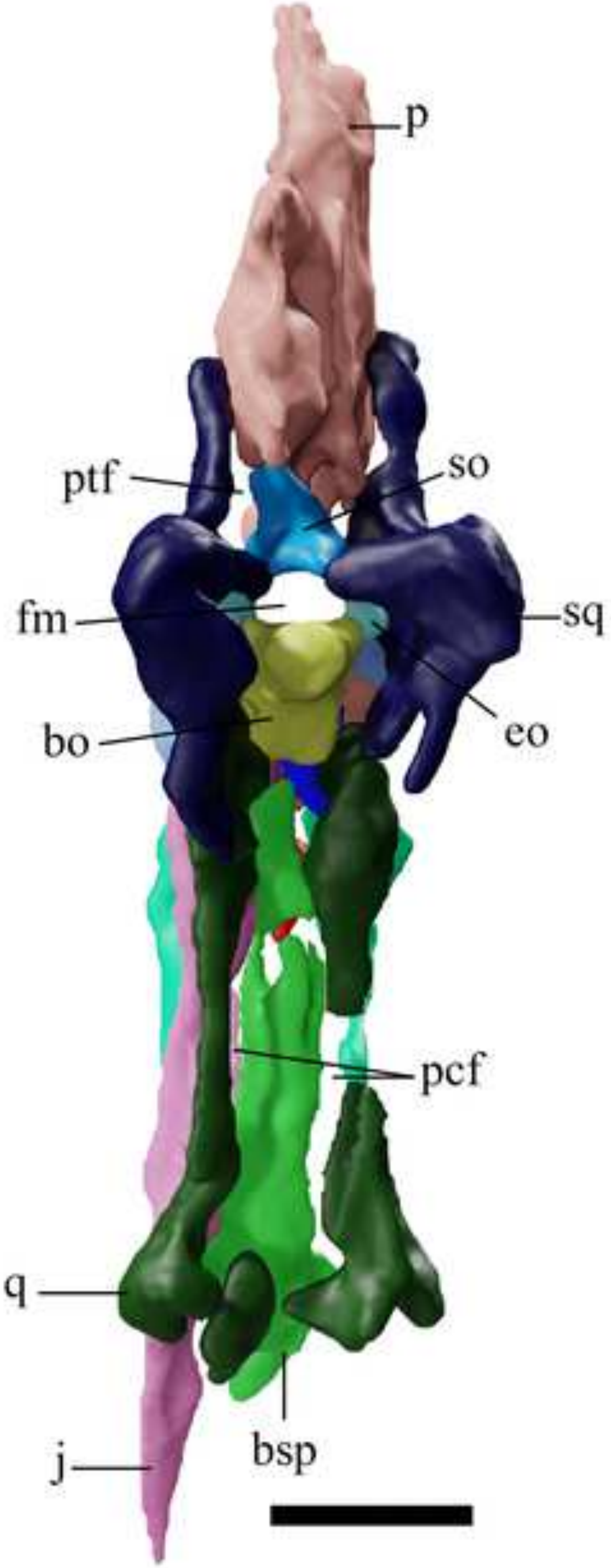

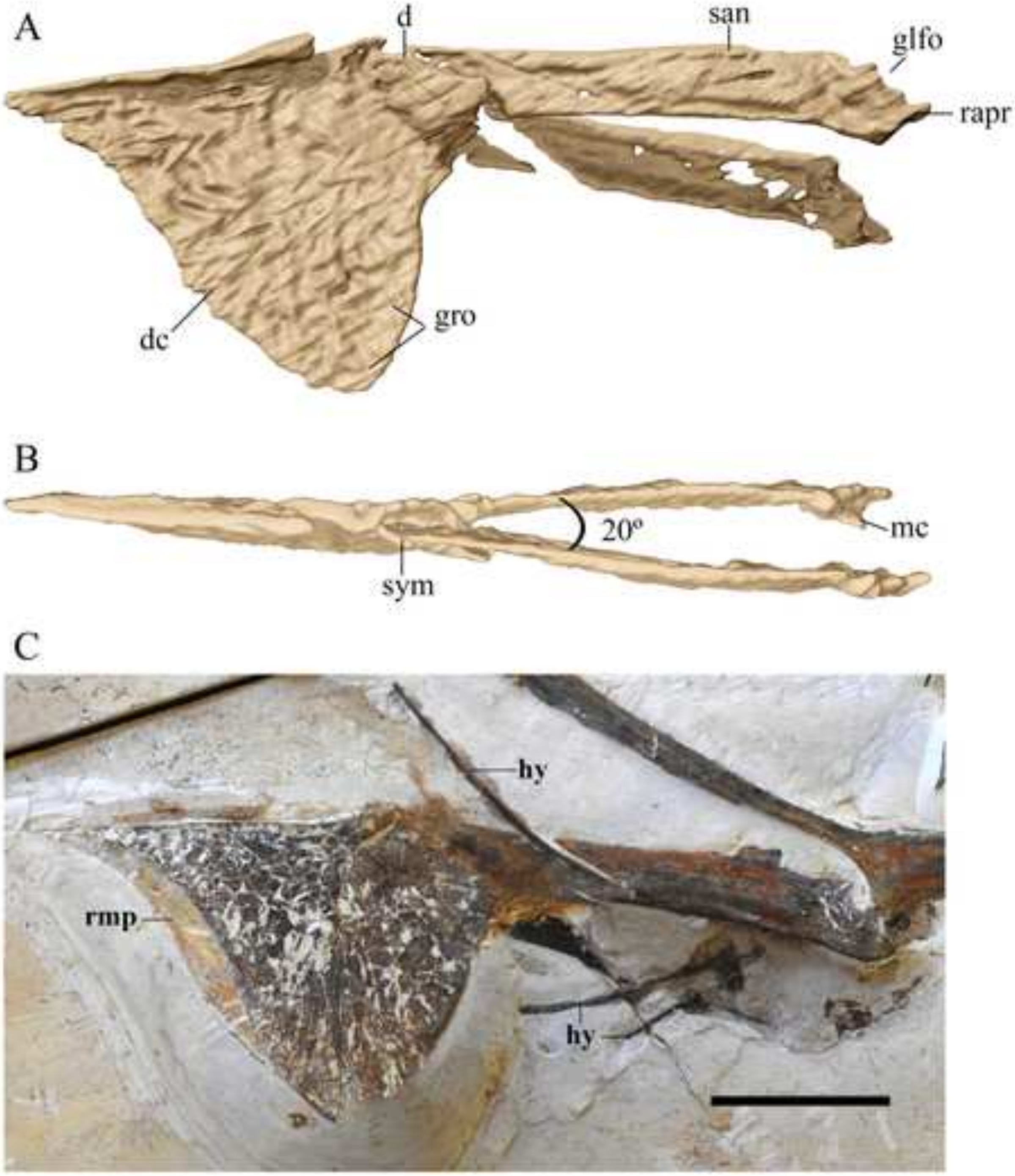
A
d
san
glfo
rapr
dc
gro
B
20º
mc
sym
C
hy
rmp
hy

Click here to access/download;Figure;Figure 5.tif

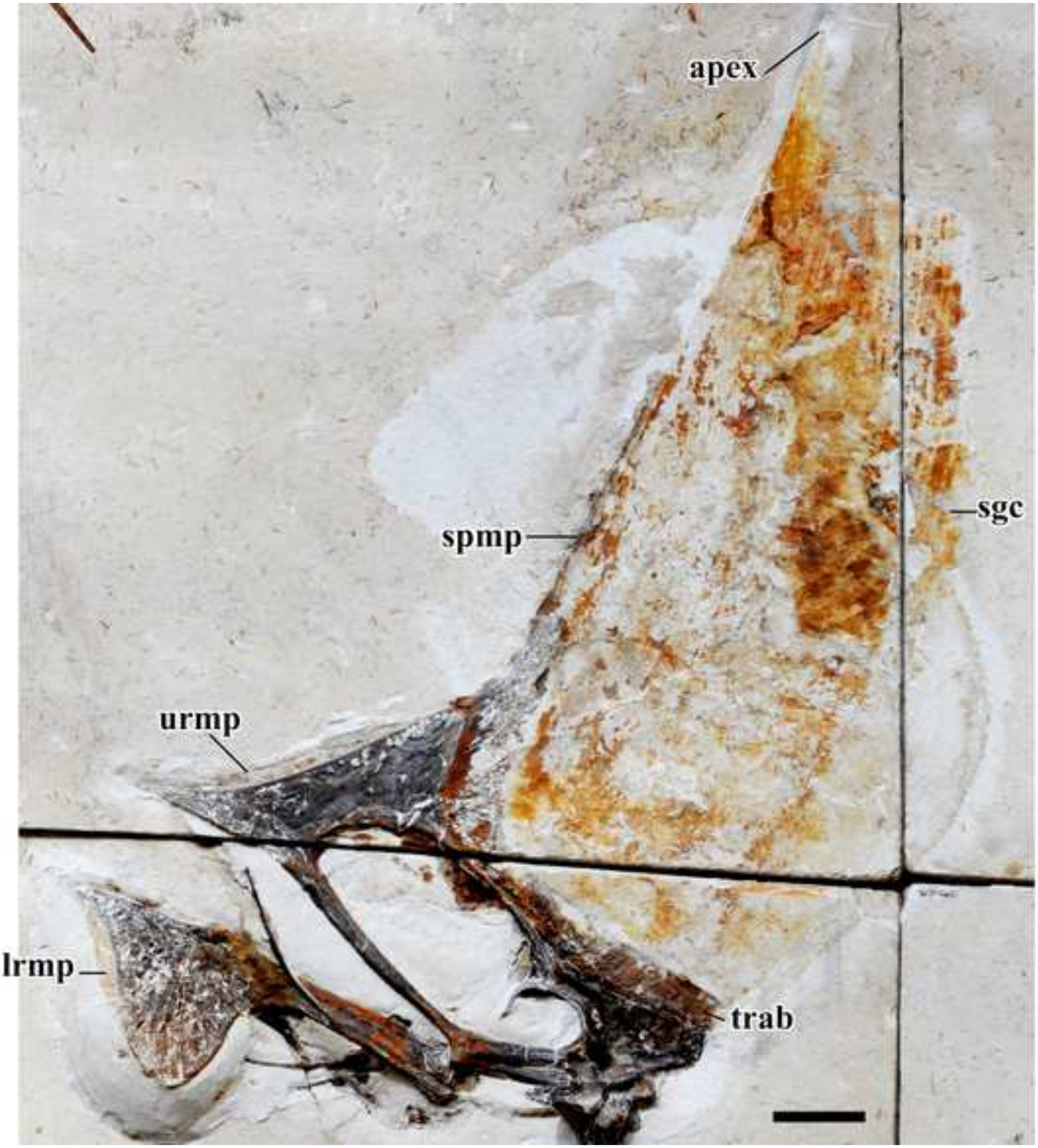

Figure 6

Click here to access/download;Figure;Figure 6.tif

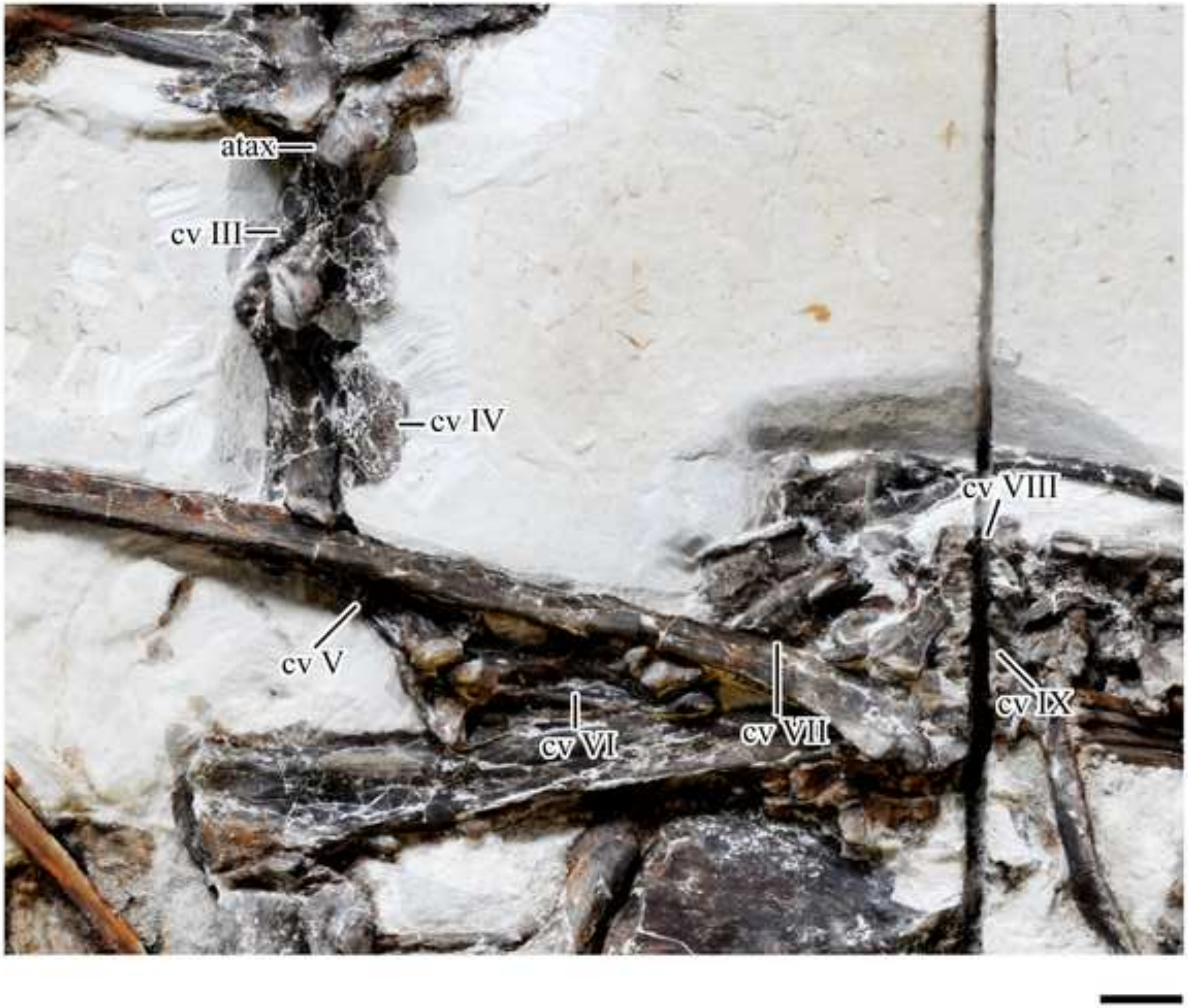

Figure 7

Click here to access/download;Figure;Figure 7.tif

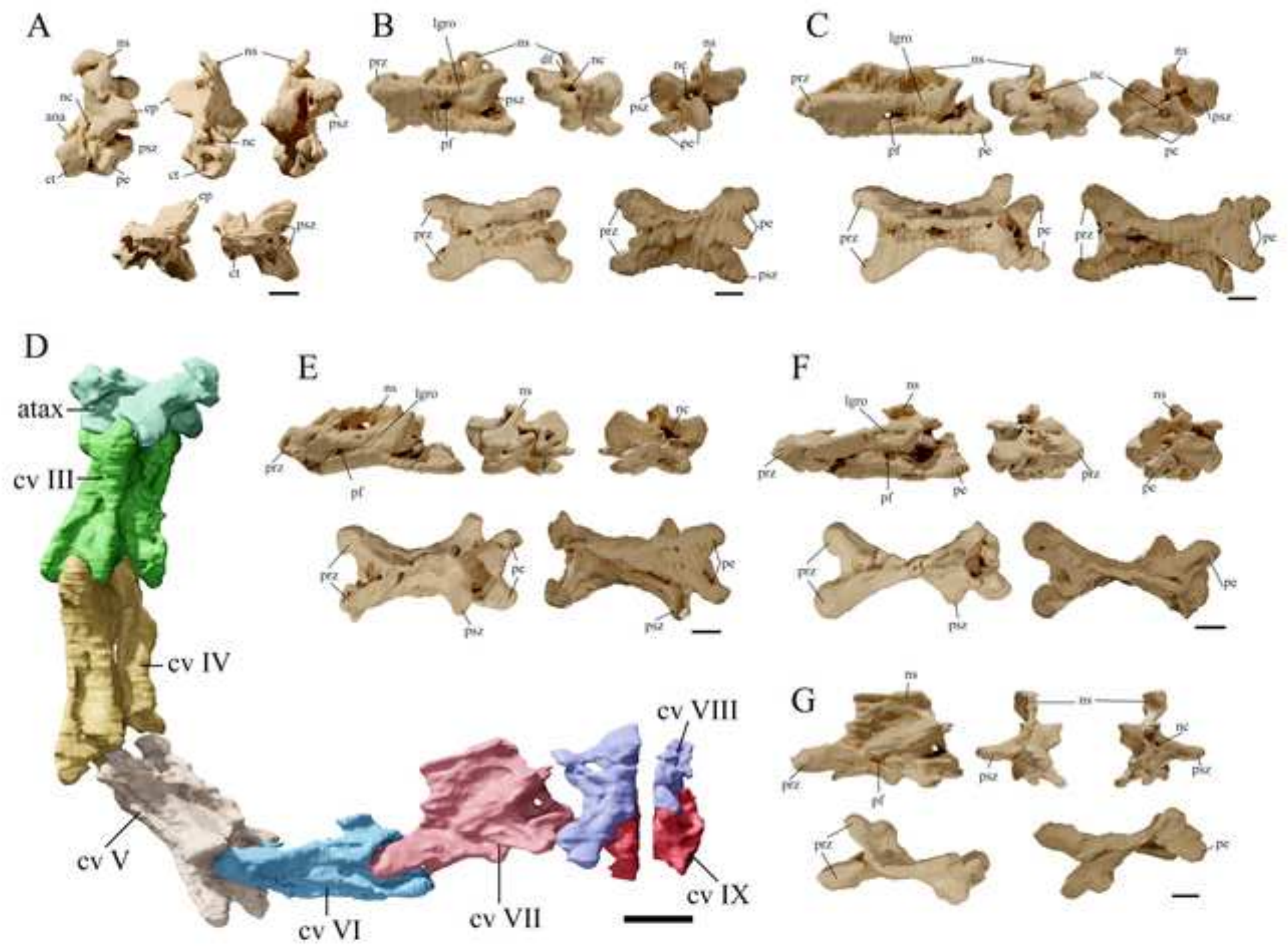

Figure 8 Click here to access/download;Figure;Figure 8.tif

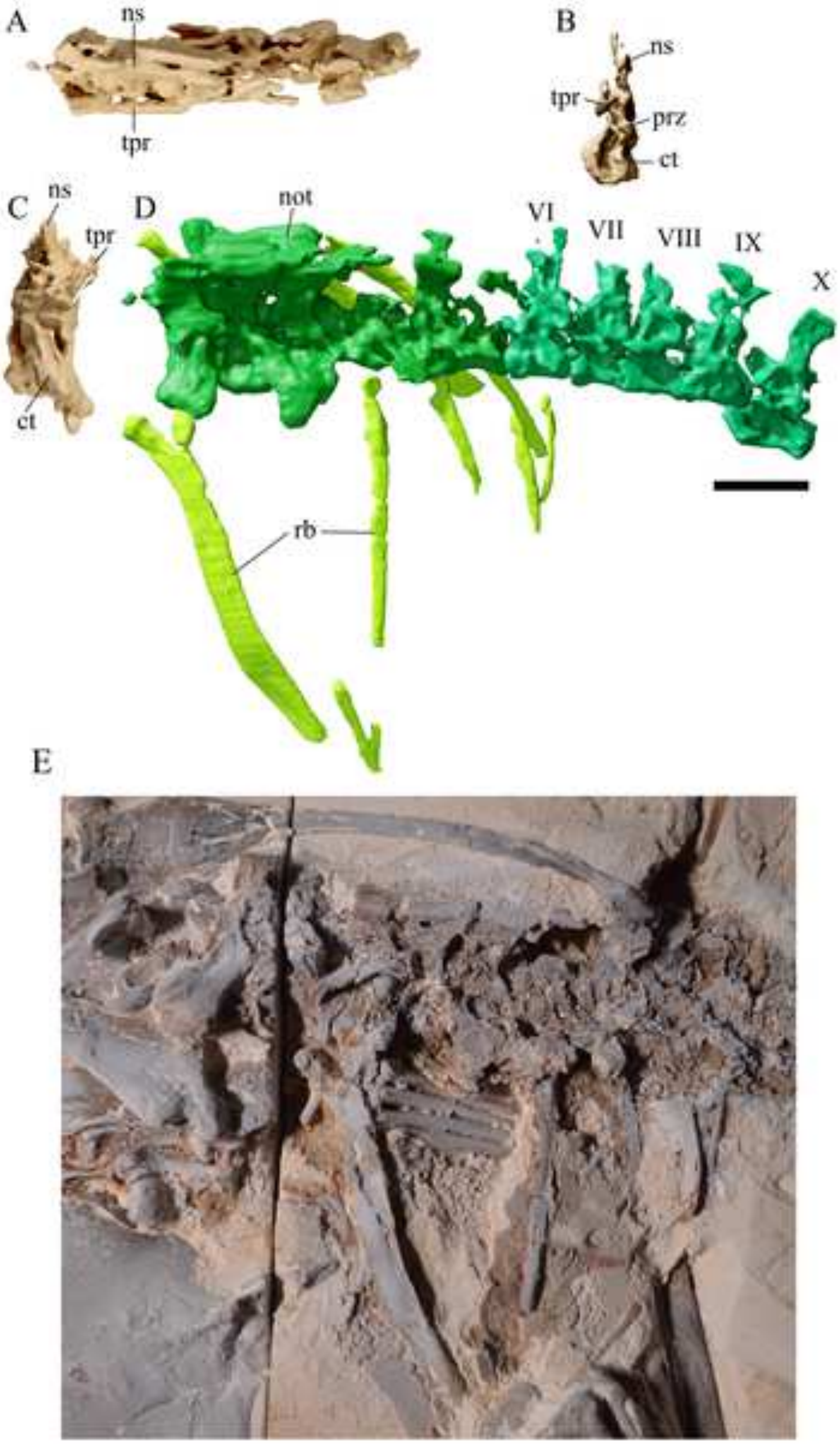

Figure 9

Click here to access/download;Figure;Figure 9.tif

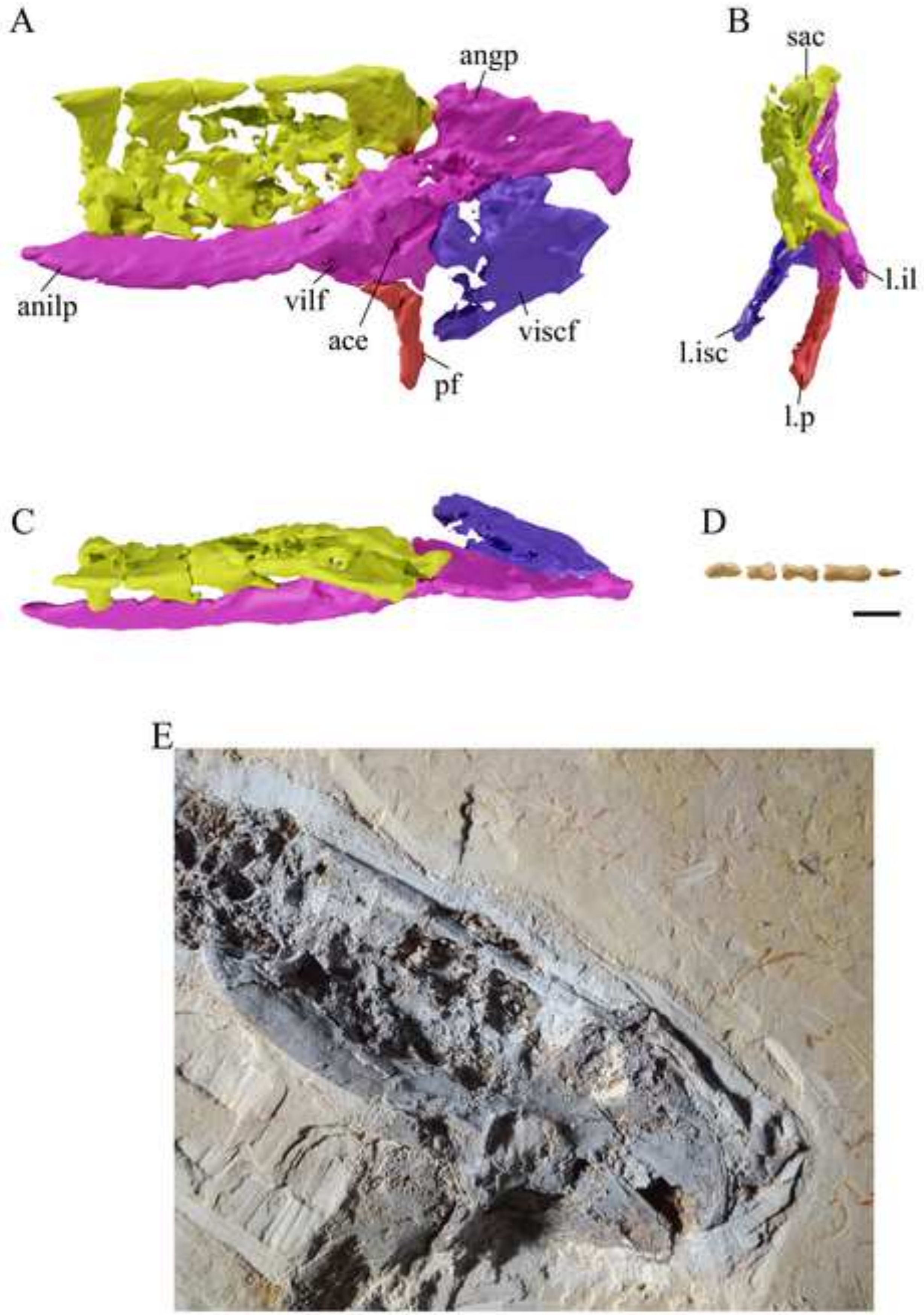

Figure 10

Click here to access/download;Figure;Figure 10.tif

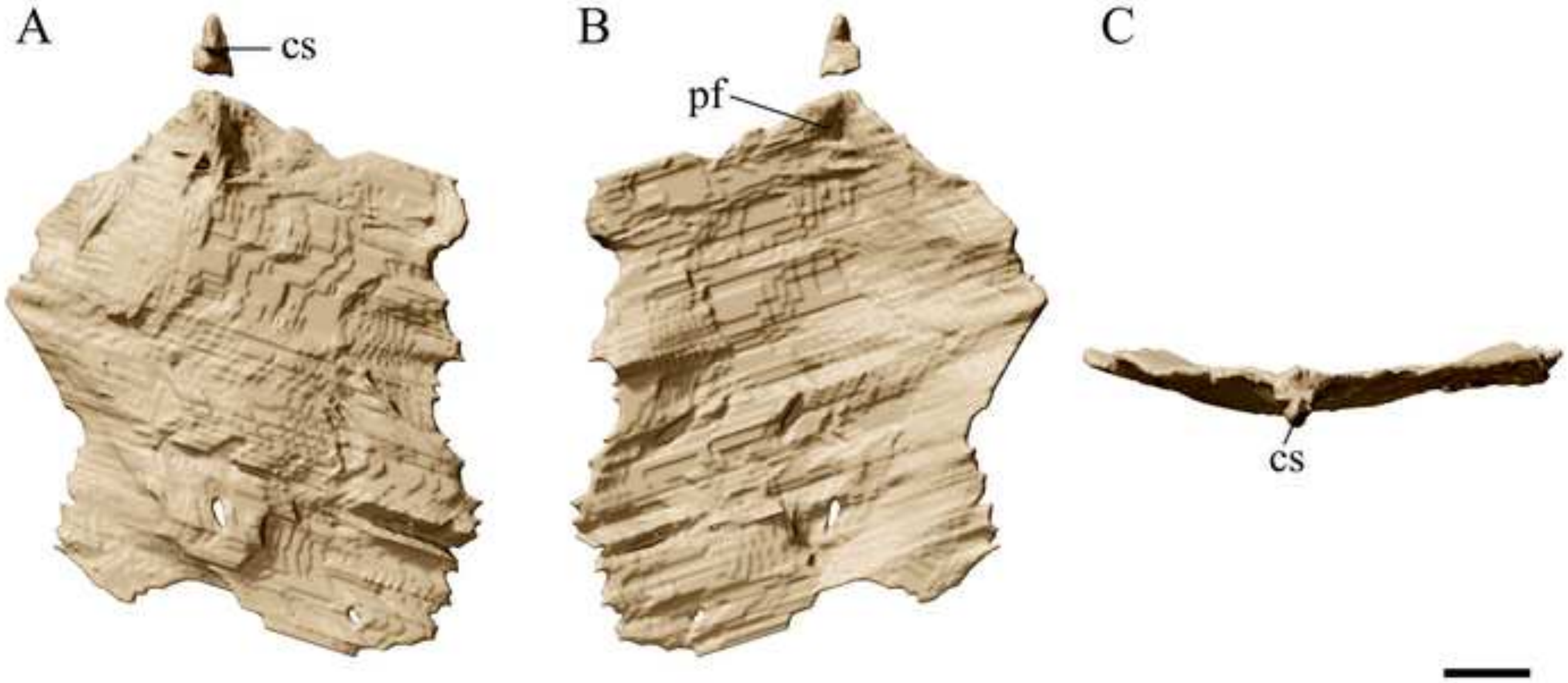

Figure 11 Click here to access/download;Figure;Figure 11.tif

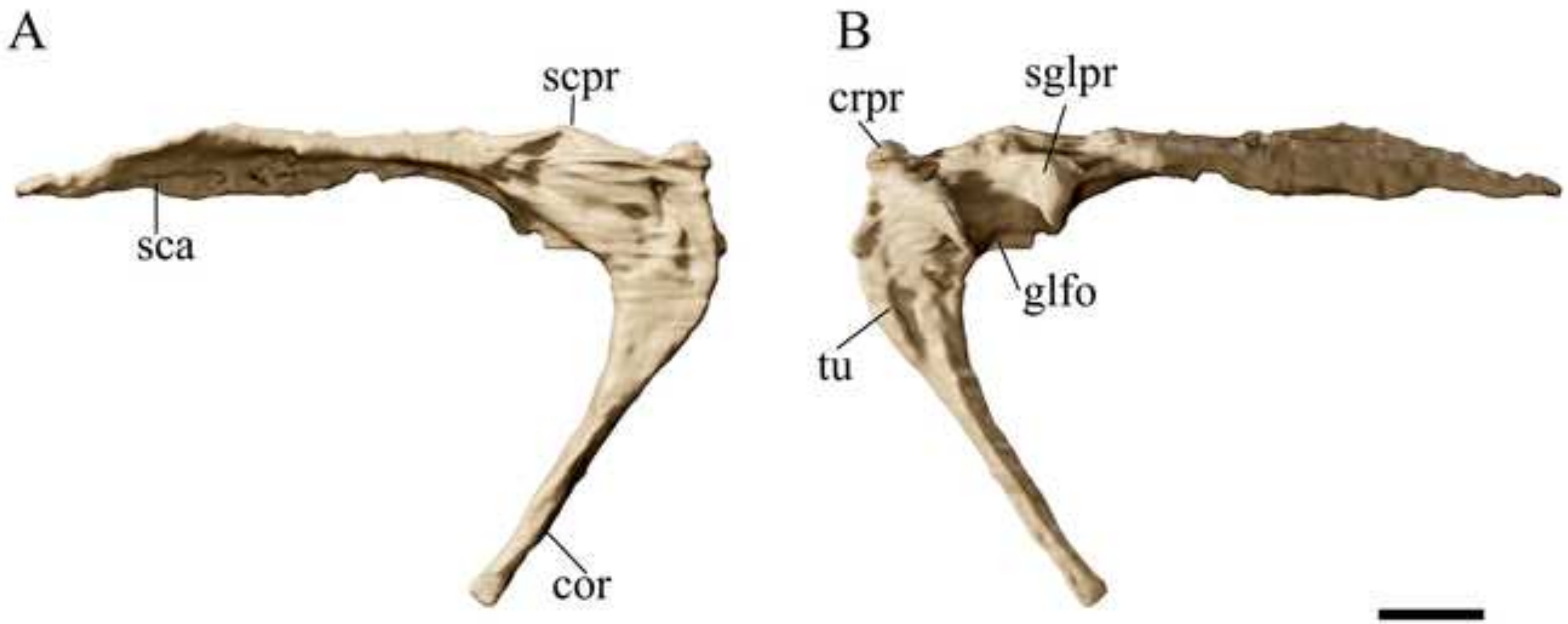

 Click here to access/download;Figure;Figure 12.tif

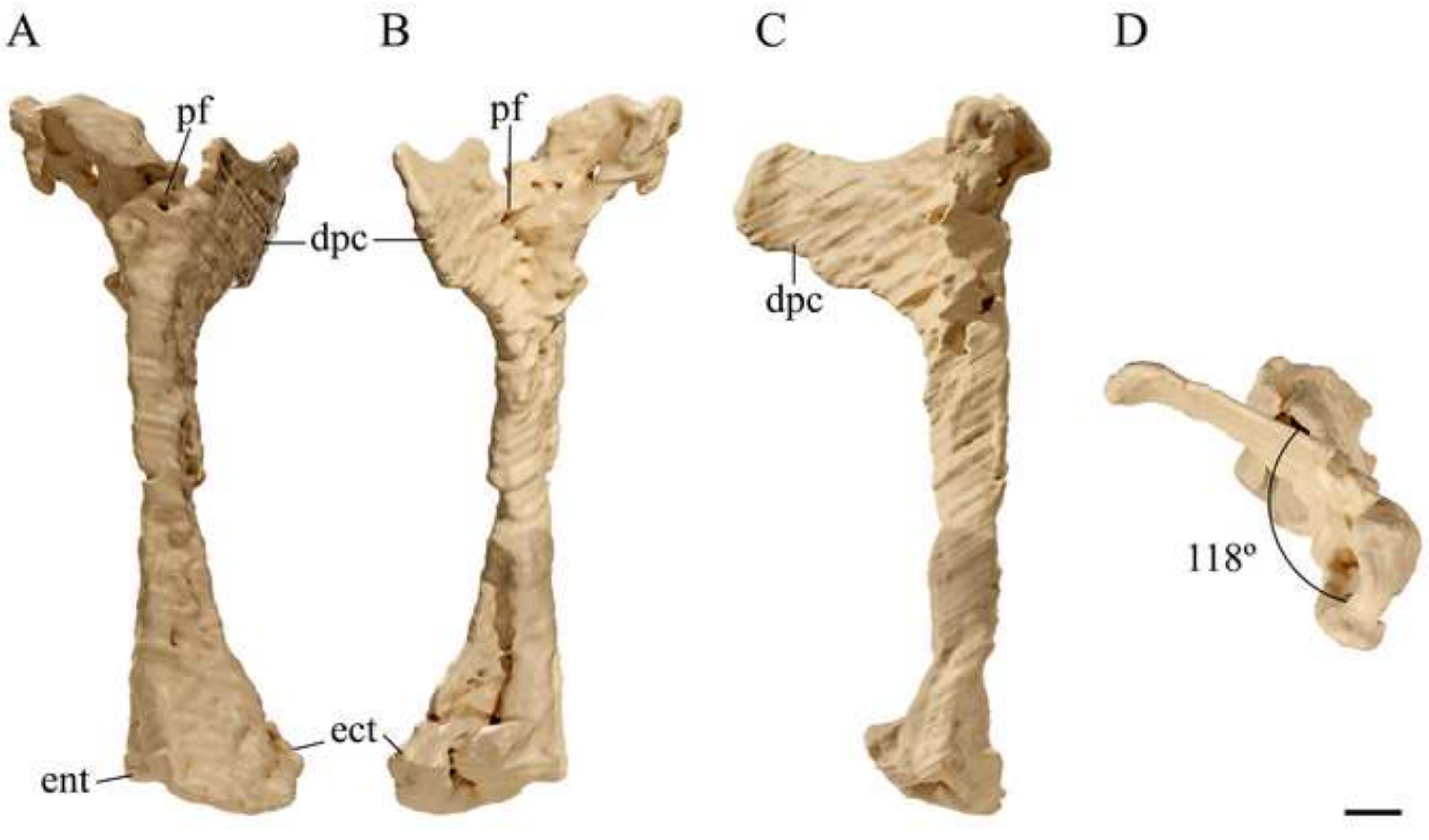

Figure 13

Click here to access/download;Figure;Figure 13.tif

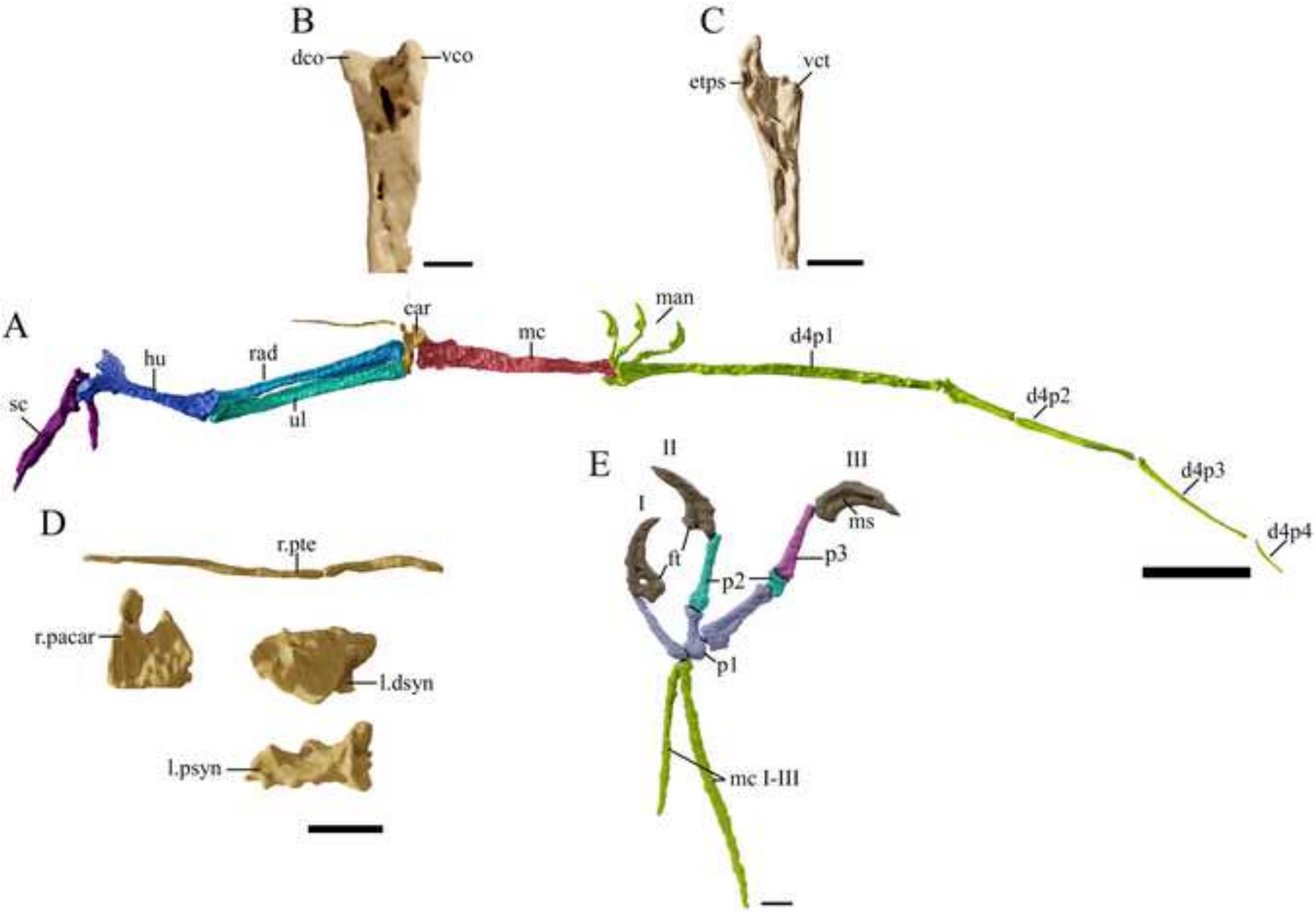

Figure 14

Click here to access/download;Figure;Figure 14.tif

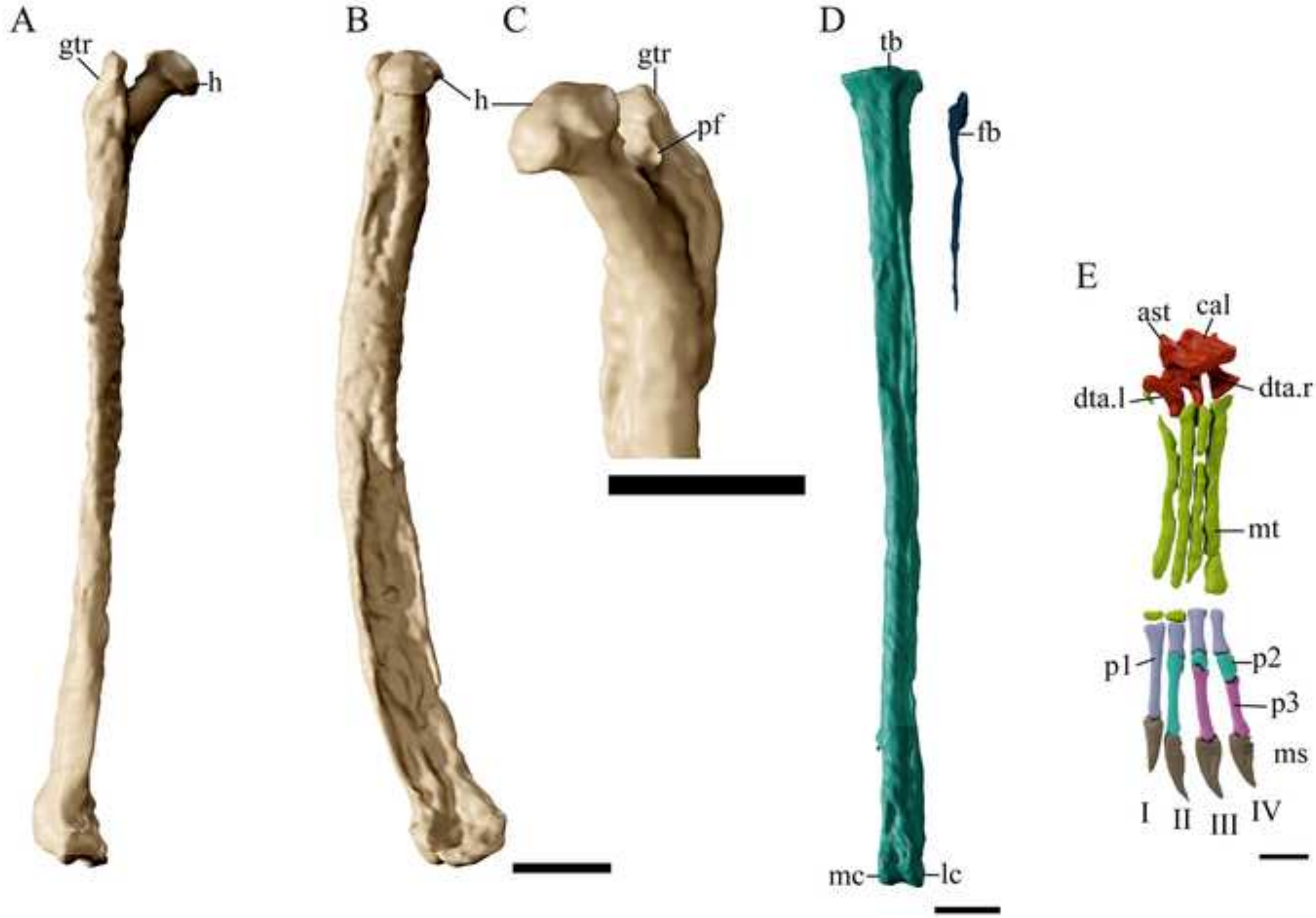

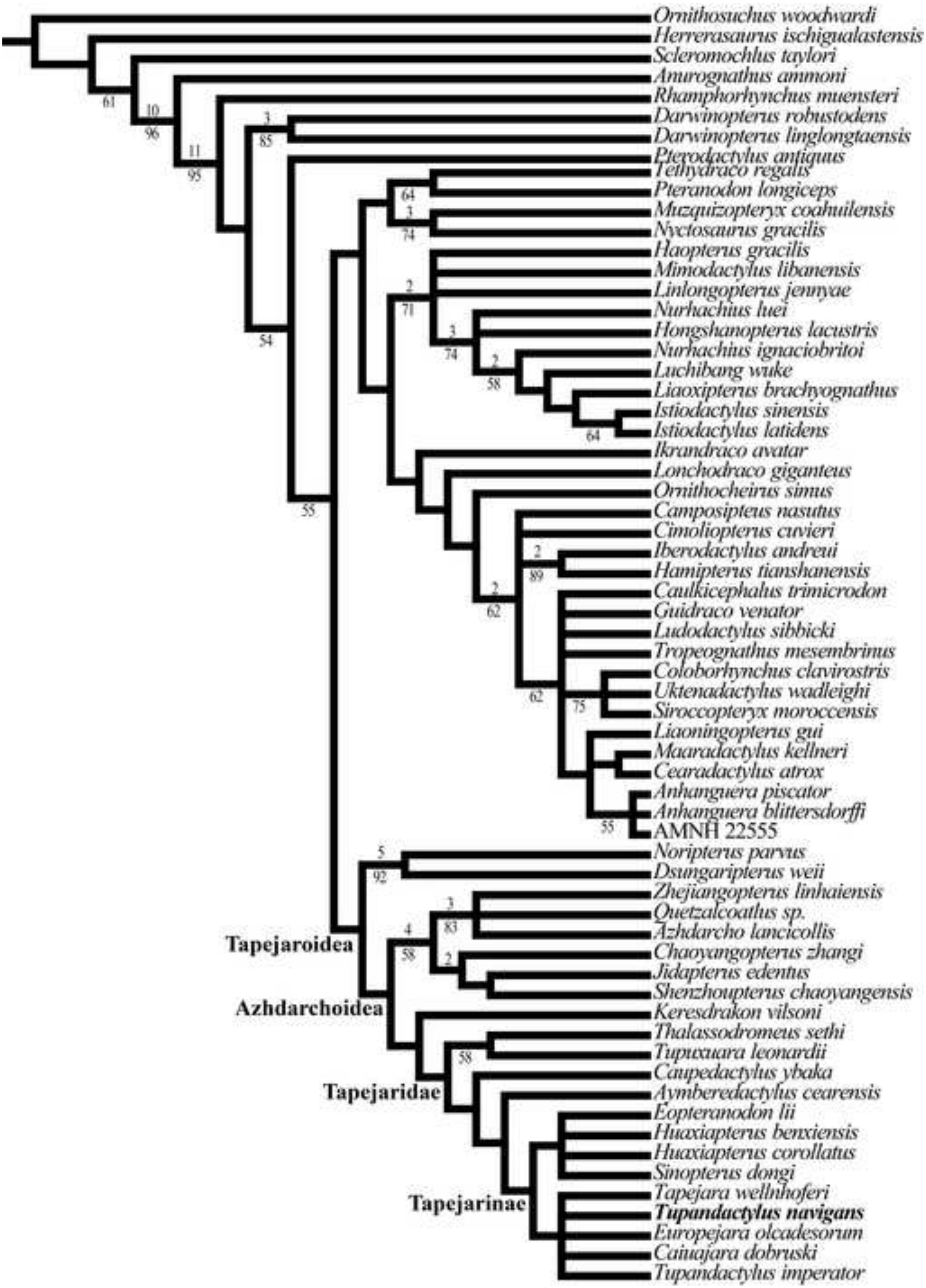
Ornithosuchus woodwardi
Herrerasaurus ischigualastensis
Scleromochlus taylori
Anurognathus ammoni
Rhamphorhynchus muensteri
Darwinopterus robustodens
Darwinopterus linglongtaensis
Pterodactylus antiquus
Tethydraco regalis
Pteranodon longiceps
Muzquizopteryx coahuilensis
Nyctosaurus gracilis
Haopterus gracilis
Mimodactylus libanensis
Linlongopterus jennyae
Nurhachius luei
Hongshanopterus lacustris
Nurhachius ignaciobritoi
Luchibang wuke
Liaoxipterus brachyognathus
Istiodactylus sinensis
Istiodactylus latidens
Ikrandraco avatar
Lonchodraco giganteus
Ornithocheirus simus
Camposipteus nasutus
Cimoliopterus cuvieri
Iberodactylus andreui
Hamipterus tianshanensis
Caulkicephalus trimicrodon
Guidraco venator
Ludodactylus sibbicki
Tropeognathus mesembrinus
Coloborhynchus clavirostris
Uktenadactylus wadleighi
Siroccopteryx moroccensis
Liaoningopterus gui
Maaradactylus kellneri
Cearadactylus atrox
Anhanguera piscator
Anhanguera blittersdorffi
AMNH 22555
Noripterus parvus
Dsungaripterus weii
Zhejiangopterus linhaiensis
Quetzalcoatlus sp.
Azhdarcho lancicollis
Chaoyangopterus zhangi
Jidapterus edentus
Shenzhoupterus chaoyangensis
Keresdrakon vilsoni
Thalassodromeus sethi
Tupuxuara leonardii
Caupedactylus ybaka
Aymberedactylus cearensis
Eopteranodon lii
Huaxiapterus benxiensis
Huaxiapterus corollatus
Sinopterus dongi
Tapejara wellnhoferi
Tupandactylus navigans
Europejara olcadesorum
Caiuajara dobruski
Tupandactylus imperator
Tapejaroidea
Azhdarchoidea
Tapejaridae
Tapejarinae
61
10
96
11
95
3
85
54
55
64
3
74
2
71
3
74
2
58
64
2
89
2
62
62
75
55
5
92
4
58
3
83
2
58

Figure 16

Click here to access/download;Figure;Figure 16.tif

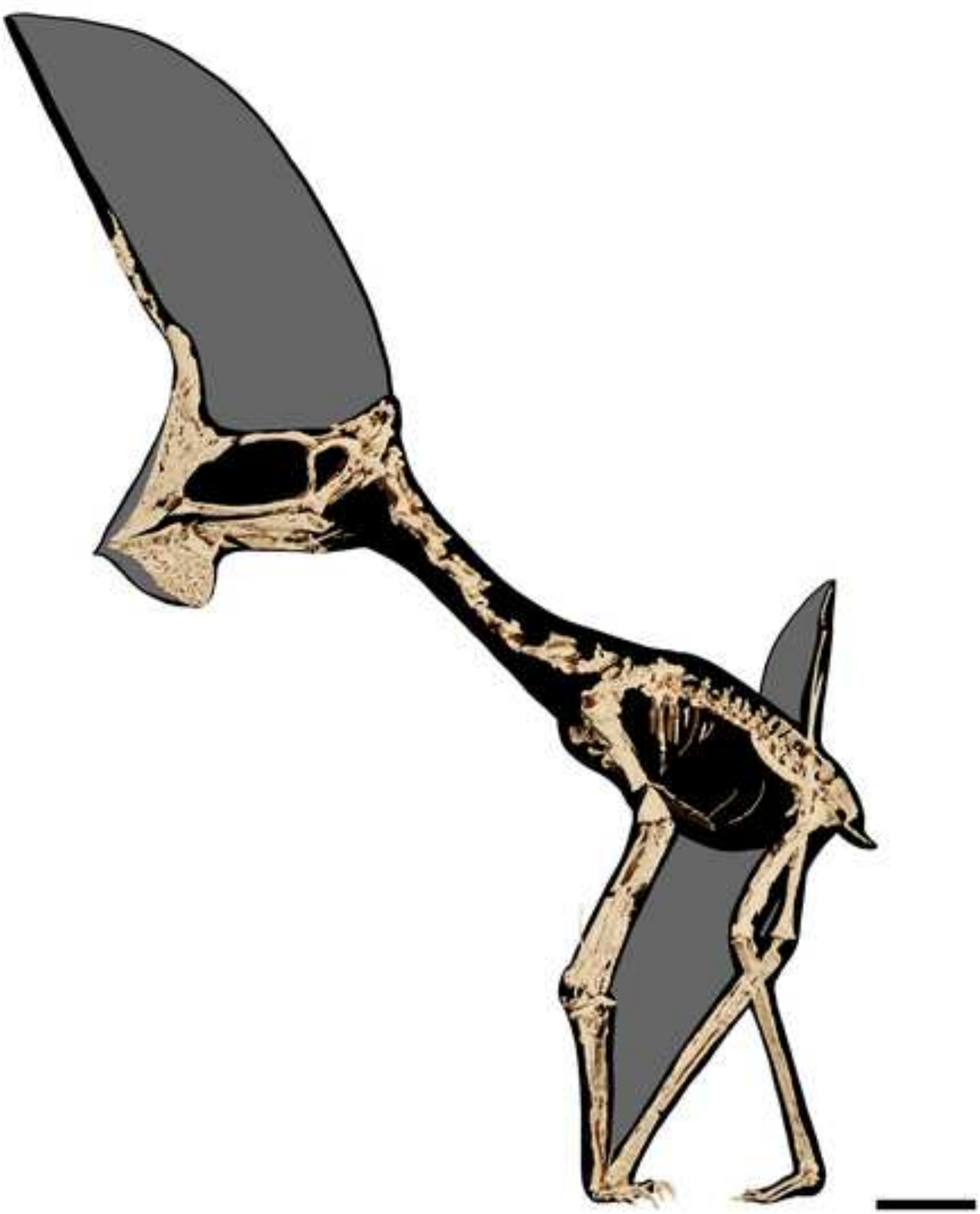

Supporting Information 1

Click here to access/download
Supporting Information
GP.2E 9266 Matrix.tnt
