# Supplementary material for: Osteology of an exceptionally well-preserved tapejarid skeleton from Brazil: Revealing the anatomy of a curious pterodactyloid clade
Source: PLoS One. 2021 Aug 25;16(8):e0254789. doi: 10.1371/journal.pone.0254789 (PMC8386889; doi:10.1371/journal.pone.0254789)
Supplement: Attachment — Submitted filename: Response to Reviewers.docx [file pone.0254789.s006.docx]

# 1 Response to the Editor Max C. Langer

All inquiries regarding the proper writing were accepted.

1 - Abbreviation of generic names and citation of species:

Please, always provide the full species name (not only the generic epithet) if this is known (otherwise use “sp.”). As for the abbreviation of the generic epithet, this is fine and welcome, but has to be patronised. So, use a full-word epithet in the first time the species is mentioned in the abstract and in the main text; after that, always use the abbreviation (unless it is the first work of a sentence). As for generic epithets starting with the same set of letters, the abbreviation has to include a minimal number of letters enough to differentiate them (e.g., Tupu. and Tupa. for Tupuxuara and Tupandactylus). I attempted to change all such cases in the MS, but please give a second look.

R: the inquiry was accepted, and the text reviewed so all species are properly written.

2 – Phylogenetic analysis:

This needs additional information for clarity and, depending on the decision of the authors, also some implementations of the analysis itself.

You wrote, “The phylogenetic position of Tupa. navigans was accessed through the scoring of GP/2E 9266 using the character-taxon matrix of Hone et al. (2020)”. As far as I could follow, this was based on Kellner et al. (2019), which was based on Holgado et al. (2019), which was based on Vullo et al. (2012), which was based on many sources. What is important to mention is [1] on what basis was Tupa. navigans scored on those previous matrices (which specimens, by which authors), and [2] if you just complemented their codification with new data from GP/2E 9266 or if you employed a completely de novo codification based only on GP/2E 9266. It seems that the latter was the case, if so, you have to clearly state if your codification is totally congruent with the previous codification of Tupa. navigans (i.e., no character was scored differently between yours and previous codifications). If this is not the case, you should consider including GP/2E 9266 as an OUT independent from Tupa. navigans (as previously scored) in those matrices; show and discuss the results. I see that this is briefly discussed in the “Phylogenetic Analysis” (apparently saying that the codifications are the same), but this needs to be clearly stated in the “M&M”, beforehand.

R: the inquiry was accepted, and the text was reviewed accordingly. “The holotype of *Tupa. navigans* SMNK PAL 2344 was initially coded as a separate OUT, but no character differed GP/2E 9266 scoring. Therefore, the phylogenetic position of *Tupa. navigans* was accessed through the scoring of GP/2E 9266 using the character-taxon matrix of [15], composed by 64 taxa (including the new specimen) and 150 discrete characters (supplementary data 1). It is worth mentioning that the dataset used derived from previous studies (i.e., [2,16,17]) and in those analysis, *Tupa. navigans* was not scored.”

You also wrote, “The second phylogenetic analysis included the specimen MN 6588-V as a separate OTU”. Firstly, you have to mention this second analysis in the “M&M” section. Secondly, it is unclear from the sentence above if MN 6588-V was previously included in the data sets as part of a given OUT or if the specimen was never considered in those data sets. You have to make this point clear.

R: the sentence in the M&M has been incremented so that it clearly states the scoring of MN 6588-V as a new OTU. “A second analysis using the same dataset and parameters was conducted including the tapejarid specimen MN 6588-V [19], a post-cranial material, as a new OTU, to assess its relationships with other tapejarids (supplementary data 2).”

Finally, you mention that the tree in Fig. 15B is a “reduced strict-consensus tree”. You have to explain how this reduced consensus tree was built in the “M&M” and then provide more detailed results, e.g., which taxa were excluded and how.

R: inquiry accepted, and a new sentence was added. “The consensus tree was recovered using the “Consensus > Strict” option of TNT v. 1.5. The reduced tree consists of the clade Tapejarinae.”

3 – Tables:

Please, do not provide them within the main text, but each in a separate file formatted and labelled accordingly.

R: due to the Journal Guidelines, the tables needed to be kept within the text.

4 – You wrote, “MN 6588-V can be regarded as Tupandactylus sp. due to the similarities to the postcranial material of GP/2E 9266”. Question: why not to Tupa. navigans, considering that GP/2E 9266 belongs to that species? In order to state that it belongs to the genus, but not to either of its species, you would have to say (if this is the case) that it does not preserve the putative differences between both species. If this is not the case, perhaps you can define the species of MN 6588-V. In any case, further explanation are needed here. See also if this leads to modifications in the conclusion.

R: inquiry accepted, and the sentence has been rewritten. “Due to the differences between MN 6588-V and GP/2E 9266 and the gap in our understanding of *Tupa. navigans* intraspecific variation, the specimen MN 6588-V cannot be confidently assigned to *Tupa. navigans.*  However, due to the abovementioned similarities to the specimen GP/2E 9266, MN 6588-V is here regarded as *Tupandactylus* sp.”

L69 - The second reason is not a good one, you can aways find a taxon in a new stratigraphic unit.

R: suggestion accepted.

L720 - What side

R: added “left”.

# 2 Response to Reviewer 2 – David W. E. Hone

5 – Comments by Dr. David Hone

Three comments by reviewer Dr. David Hone were not clearly tacked in the modified MS. Please respond to these queries: [1] about the hook-like structure of the rhamphotheca, Dr. Hone asked “I don't understand this at all. It doesn't look like it is hooked and I do not know how a 'peg' would operate. Please explain.”;

R: this was already changed but overlooked in the “response to reviewers”. The inquiry was accepted, and the sentence changed. “In pterosaurs such as *T. navigans*, the rhamphotheca could have been a hook-like structure operating to catch or to manipulate small food items [78].”

[2] In the sentence “wing phalanges length compared to the inner-wing length in GP/2E 9266 is comparatively short” Dr. Hone asked for companions;

R: this section of the text was restructured. “Ultimately, the first wing phalanx length compared to metacarpal IV length in GP/2E 9266 is comparatively short (first wing phalanx length to metacarpal IV length in GP/2E 9266 = 0.58. For anhanguerids, this ratio is closer to 0.4 [64]).”

[3] About the sentence “Frey and colleagues argued that, in order to have an aerodynamically functional crest … ”, Dr. Hone also commented that “they assume crests were functional which is very unlikely (see Hone et al. 2010). If crests were functional we'd expect them to converge on a very small number of forms, instead they show very high diversity which is a signature of socio-sexually structures”. Please respond to his query.

R: the inquiry was accepted, and a new sentence was written to accommodate the reviewers note. “Although the crests in pterosaurs were possibly used as social and/or sexual features (see [80]), Frey and colleagues [9] argued that, in order to have an aerodynamically functional crest, *Tupa. navigans* should have had a short neck or tendon locks on its cervical vertebrae.”
